# FAK and Pyk2 activity promote TNF-α and IL-1β-mediated pro-inflammatory gene expression and vascular inflammation

**DOI:** 10.1038/s41598-019-44098-2

**Published:** 2019-05-20

**Authors:** James M. Murphy, Kyuho Jeong, Yelitza A. R. Rodriguez, Jung-Hyun Kim, Eun-Young Erin Ahn, Ssang-Taek Steve Lim

**Affiliations:** 1 Department of Biochemistry and Molecular Biology, College of Medicine, University of South Alabama, Mobile, AL 36688 USA; 2 Mitchell Cancer Institute, University of South Alabama, Mobile, AL 36604 USA

**Keywords:** Extracellular signalling molecules, Atherosclerosis

## Abstract

Protein tyrosine kinase (PTK) activity has been implicated in pro-inflammatory gene expression following tumor necrosis factor-α (TNF-α) or interkeukin-1β (IL-1β) stimulation. However, the identity of responsible PTK(s) in cytokine signaling have not been elucidated. To evaluate which PTK is critical to promote the cytokine-induced inflammatory cell adhesion molecule (CAM) expression including VCAM-1, ICAM-1, and E-selectin in human aortic endothelial cells (HAoECs), we have tested pharmacological inhibitors of major PTKs: Src and the focal adhesion kinase (FAK) family kinases - FAK and proline-rich tyrosine kinase (Pyk2). We found that a dual inhibitor of FAK/Pyk2 (PF-271) most effectively reduced all three CAMs upon TNF-α or IL-1β stimulation compared to FAK or Src specific inhibitors (PF-228 or Dasatinib), which inhibited only VCAM-1 expression. *In vitro* inflammation assays showed PF-271 reduced monocyte attachment and transmigration on HAoECs. Furthermore, FAK/Pyk2 activity was not limited to CAM expression but was also required for expression of various pro-inflammatory molecules including MCP-1 and IP-10. Both TNF-α and IL-1β signaling requires FAK/Pyk2 activity to activate ERK and JNK MAPKs leading to inflammatory gene expression. Knockdown of either FAK or Pyk2 reduced TNF-α-stimulated ERK and JNK activation and CAM expression, suggesting that activation of ERK or JNK is specific through FAK and Pyk2. Finally, FAK/Pyk2 activity is required for VCAM-1 expression and macrophage recruitment to the vessel wall in a carotid ligation model in *ApoE*−/− mice. Our findings define critical roles of FAK/Pyk2 in mediating inflammatory cytokine signaling and implicate FAK/Pyk2 inhibitors as potential therapeutic agents to treat vascular inflammatory disease such as atherosclerosis.

## Introduction

The vascular endothelium plays a key role in directing the immune response to tissues experiencing an inflammatory insult. Several diseases that affect the endothelium, such as atherosclerosis, hypertension, and diabetes, result in chronic inflammation of the vascular endothelium^1,2^. Common vascular insults, such as hypercholesterolemia, high blood pressure, and reactive oxygen species, associated with these diseases lead to activation of endothelial cells (ECs) and resident leukocytes of the vessel wall^2–9^. These activated cells then promote secretion of major pro-inflammatory cytokines such as tumor necrosis factor-α (TNF-α) and interleukin-1β (IL-1β)^10–12^. Pro-inflammatory cytokines stimulate the ECs and induce expression of cell adhesion molecules (CAMs) and chemokines which recruit blood-borne leukocytes to the vessel wall. In the case of atherosclerosis, the recruited monocytes differentiate into macrophages and eventually become lipid-laden foam cells in chronic hypercholesterolemia conditions^13,14^. Studies have shown that pharmacological approaches, such as lipid lowering and antihypertensive drugs, have helped reduce the risk of cardiovascular diseases associated with atherosclerosis^15–17^. However, there are currently no therapies that block the endothelium response to inflammatory cytokines.

While several models have been developed to study vascular inflammation *in vitro*, such as monocyte attachment and trans-endothelial migration assays, have been developed^18^, *in vivo* models offer a better system in which to study the effect of treatments on vascular inflammation. Animal models mimicking human atherosclerosis are one of the best characterized models dealing with vascular inflammation. Apolipoprotein E (ApoE) and low-density lipoprotein receptor (LDLR) knockout mice fed a high fat diet develop chronic inflammation of the vessel wall leading to EC dysfunction, inflammatory CAM expression, and macrophage recruitment^19–21^. Elevated inflammatory cytokines such as TNF-α and IL-1β have been found in both human and animal atherosclerotic lesions^22,23^, and knockdown of these cytokines have alleviated atherosclerosis progression^24,25^. These studies suggest blocking inflammatory cytokine signaling as a potential target in the treatment of vascular inflammation.

Both TNF-α and IL-1β activate mitogen-activated protein kinases (MAPKs) and nuclear factor-κB (NF-κB) signaling pathways to induce expression of inflammatory molecules^26,27^. In chronic inflammatory diseases, continued exposure to inflammatory stimuli leads to elevated activation of these pathways and disrupting these pathways has been implicated in treating inflammatory diseases. Interestingly, both TNF-α and IL-1β lead to rapid tyrosine phosphorylation in various cell types, and protein tyrosine kinases (PTKs) have been implicated as potential targets to block pro-inflammatory signaling cascades^28^. Inhibition of PTKs with nonselective tyrosine kinase inhibitors (e.g., genistin and herbimycin A) has been shown to reduce TNF-α or IL-1β-induced CAM expression^29^. Important PTKs such as Src, and the focal adhesion kinase (FAK) family kinases - FAK and proline-rich tyrosine kinase (Pyk2) - have been implicated in the activation of MAPKs and NF-κB, as well as in the expression of CAMs in response to both TNF-α and IL-1β^30–37^. However, which PTK(s) plays a major role in these signaling pathways remains unknown.

An emerging body of evidence suggests that FAK, an integrin-associated PTK, might play a major role in inflammatory cytokine signaling. FAK expression is critical to regulate TNF-α downstream signaling through its association with receptor-interacting serine/threonine-protein kinase 1 (RIPK1) and TNF receptor-associated factor 2 (TRAF2)^34,38^. TNF-α-mediated IL-6 expression requires FAK activity to activate MAPK signaling^35^. FAK also contributes to IL-1β signaling pathway by associating with protein-tyrosine phosphatase-α (PTPα) to activate MAPK and to enhance matrix metalloproteinase-9 (MMP-9) expression^39,40^. FAK activity controls vascular cell adhesion molecule-1 (VCAM-1) and E-selectin in mouse fibroblasts and cancer cells^41,42^. However, it is not known how FAK activity contribute to TNF-α and IL-1β signaling that promotes cell adhesion molecule expression in ECs.

In the present study, we have investigated the contribution of PTK activity through the use of small molecule pharmacological inhibitors against FAK, Pyk2, and Src in the regulation of CAM expression upon TNF-α or IL-1β stimulation in human ECs. We found that dual inhibition of FAK/Pyk2 activity was the most effective at blocking cytokine-induced inflammatory molecule expression through ERK and JNK MAPK signaling. Furthermore, inhibition of FAK/Pyk2 activity reduced monocyte attachment and trans-endothelial migration *in vitro*, as well as VCAM-1 expression and macrophage recruitment in an *ApoE*−/− mouse model of inflammation. These studies reveal a therapeutic potential of pharmacological FAK/Pyk2 inhibitors in the treatment of vascular inflammatory diseases and atherosclerosis.

## Results

### FAK, Pyk2, and Src differentially regulate TNF-α-induced inflammatory cell adhesion molecule expression in human endothelial cells

**Figure 1:**
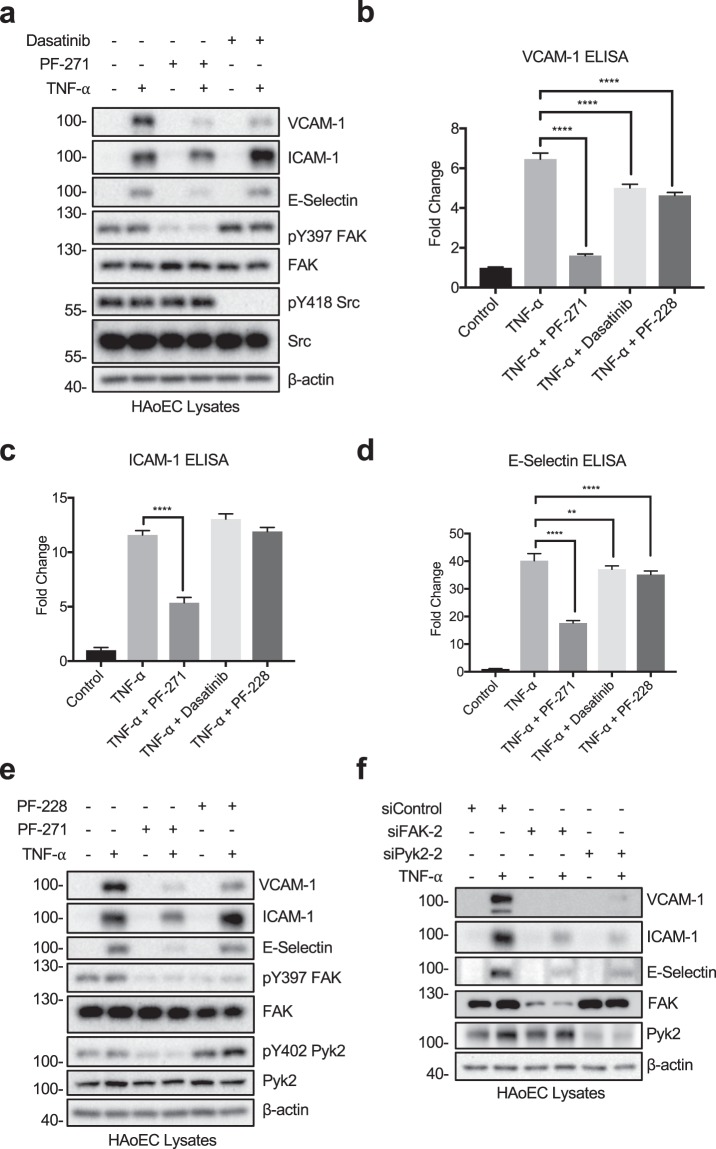
FAK/Pyk2 inhibition blocks TNF-α-induced pro-inflammatory adhesion molecule expression in HAoECs. (**a**) HAoECs were treated with DMSO, a dual FAK/Pyk2 inhibitor (PF-271, 2.5 μM) or a Src inhibitor (Dasatinib, 1 μM) for 1 h prior to TNF-α (10 ng/ml, 6 h) stimulation. Cropped images of immunoblotting for VCAM-1, ICAM-1, E-selectin, active FAK (pY397 FAK), FAK, active Src (pY418 Src), Src, and β-actin as loading control are shown. Full length blots shown in Supplemental Fig. 7. (**b**–**d**) HAoECs were treated with DMSO, a dual FAK/Pyk2 inhibitor (PF-271, 2.5 μM), a Src inhibitor (Dasatinib, 1 μM), or a FAK inhibitor (PF-228, 10 μM) for 1 h prior to TNF-α (10 ng/ml, 6 h) stimulation. Expression levels of (**b**) VCAM-1, (**c**) ICAM-1, and (**d**) E-selectin were determined using ELISA (n = 3, ±SEM). (**e**) HAoECs were treated with DMSO, a dual FAK/Pyk2 inhibitor (PF-271, 2.5 μM) or a FAK inhibitor (PF-228, 10 μM) for 1 h prior to TNF-α (10 ng/ml, 6 h) stimulation. Cropped images of immunoblotting for VCAM-1, ICAM-1, E-selectin, pY397 FAK, FAK, pY402 Pyk2, Pyk2, and β-actin as loading control are shown. Full length blots shown in Supplemental Fig. 8. (**f**) HAoECs were transfected with either control, FAK siRNA (400 pmol, 36 h), or Pyk2 siRNA (400 pmol, 36 h) prior to TNF-α (10 ng/ml, 4 h) stimulation. Cropped images of immunoblotting for VCAM-1, ICAM-1, E-selectin, FAK, Pyk2, and β-actin as loading control are shown. Full length blots shown in Supplemental Fig. 9. **p < 0.01, ****p < 0.0001.

To determine which protein tyrosine kinases (PTKs) contribute to the expression of CAMs upon TNF-α stimulation, human aortic endothelial cells (HAoECs) were pretreated with either PF-271 or Dasatinib prior to TNF-α stimulation. Inhibition of FAK and Src was verified by performing active marker blotting with pY397 FAK and pY418 Src antibodies (Fig. 1a). PF-271 decreased TNF-α-stimulated VCAM-1 expression (Fig. 1a,b and Supp. Fig. 1a,b). Moreover, PF-271 also reduced the expression of two other key inflammatory CAMs, intercellular adhesion molecule-1 (ICAM-1) and E-selectin, in HAoECs (Fig. 1a,c,d and Supp. Fig. 1b). In contrast, Dasatinib only reduced expression of VCAM-1, but did not affect expression of ICAM-1 and E-selectin (Fig. 1a–d and Supp. Fig. 1b). These data suggest that FAK family kinases are key PTKs affecting TNF-α-stimulated pro-inflammatory CAM expression.

Since PF-271 can inhibit both FAK and Pyk2, it is unclear which kinase is important for TNF-α-mediated inflammatory CAM expression in human ECs. In order to separate FAK and Pyk2 signaling, we next compared PF-271 with a FAK specific inhibitor (PF-228) in TNF-α-induced CAM expression. As expected, treatment with PF-271 or PF-228 reduced active pY397 FAK levels (Fig. 1e). As FAK specific inhibitors increase Pyk2 activity^43^, we next evaluated active pY402 Pyk2 levels. While treatment with PF-271 reduced pY402 Pyk2, PF-228 induced a compensatory increase in pY402 Pyk2 activity in HAoECs (Fig. 1e). PF-271 reduced expression of all three CAMs, but inhibition with PF-228 only reduced expression of VCAM-1, suggesting that blocking Pyk2 activity may also be required for effectively reducing TNF-α-induced ICAM-1 and E-selectin expression (Fig. 1b–e and Supp. Fig. 1c,d). Another dual FAK/Pyk2 pharmacological inhibitor (VS-6063) currently in development as a cancer therapy^44^ also efficiently reduced VCAM-1, ICAM-1, and E-selectin expression in a dose dependent manner in HAoECs (Supp. Fig. 1e), suggesting that inhibition of both FAK and Pyk2 simultaneously is required to reduce inflammatory CAM expression.

Since there is currently no Pyk2 specific small molecule inhibitor available, we used small interfering RNA (siRNA) to knockdown either FAK or Pyk2 to further evaluate their specific role in TNF-α-induced CAM expression. FAK or Pyk2 knockdown was confirmed by total FAK or Pyk2 immunoblotting (Fig. 1f and Supp. Fig. 2). Interestingly, both siFAK and siPyk2 reduced expression of VCAM-1, ICAM-1, and E-selectin (Fig. 1f and Supp. Fig. 2). As FAK family kinases exhibit both kinase-dependent and -independent functions in various cellular signaling pathways^45^, expression of both FAK and Pyk2 may be a prerequisite for pro-inflammatory signaling and maximal CAM expression.

### FAK, Pyk2, and Src inhibition differentially regulate TNF-α-induced MAPK activation in human endothelial cells

**Figure 2:**
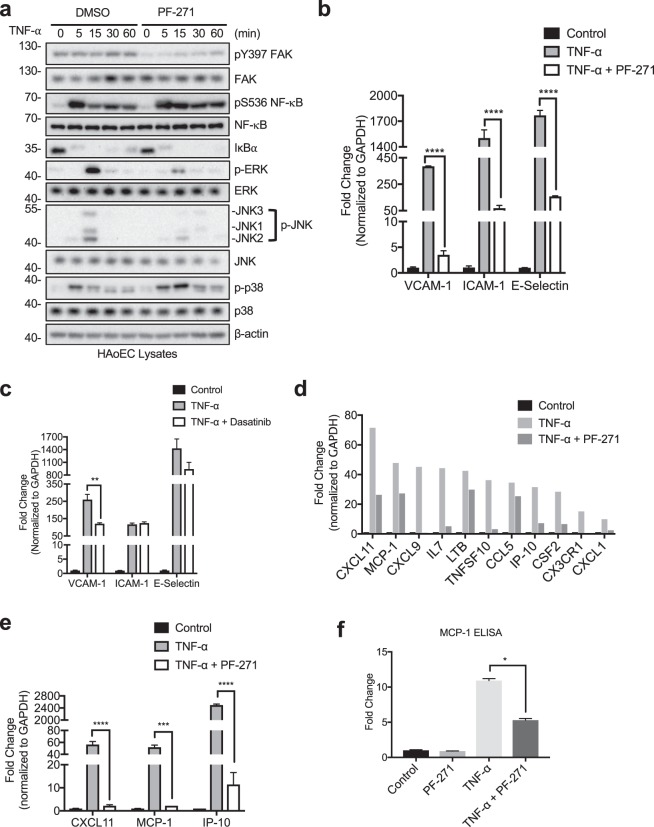
FAK/Pyk2 activity promotes TNF-α-mediated pro-inflammatory molecule expression via transcriptional control. (**a**) HAoECs were treated with DMSO or PF-271 (2.5 μM) for 1 h prior to TNF-α (10 ng/ml) stimulation for 0 to 60 min. Cropped images of immunoblotting for pY397 FAK, FAK, active NF-κB (pS536 NF-κB), NF-κB, IκBα, active ERK (p-ERK), ERK, active JNK (p-JNK), JNK, active p38 (p-p38), p38, and β-actin as loading control are shown. Full length blots shown in Supplemental Fig. 10. (**b**) HAoECs were treated with DMSO or PF-271 (2.5 μM) for 1 h prior to TNF-α (10 ng/ml, 6 h) stimulation. RNA was collected, and RT-qPCR for cell adhesion molecules was performed (n = 3, ±SEM). (**c**) HAoECs were treated with DMSO or Dasatinib (1 μM) for 1 h prior to TNF-α (10 ng/ml, 6 h) stimulation. RNA was collected, and RT-qPCR for cell adhesion molecules was performed (n = 3, ±SEM). (**d**) HAoECs were treated with DMSO or PF-271 (2.5 μM) for 1 h prior to TNF-α (10 ng/ml, 24 h) stimulation. RNA was collected, and RT-qPCR was performed using an array for inflammatory cytokines, chemokines and their receptors. Shown is a selection of genes that is important in vascular inflammation and were reduced by PF-271 treatment upon TNF-α stimulation. (**e**) CXCL11, MCP-1, and IP-10 mRNA levels were verified by RT-qPCR (n = 3, ±SEM). (**f**) HAoECs were treated with DMSO or PF-271 (2.5 μM) for 1 h prior to TNF-α (10 ng/ml, 24 h) stimulation. Supernatant was collected, and MCP-1 protein expression was determined via ELISA (n = 3, ±SEM). *p < 0.05, **p < 0.01, ***p < 0.001, ****p < 0.0001.

As TNF-α stimulation induces expression of inflammatory molecules through activation of MAPKs and NF-κB signaling pathways, we tested if FAK/Pyk2 activity affects these pathways by performing a 0 to 60 min time course experiment. PF-271 treatment reduced activation of both ERK and JNK but slightly increased p38 MAPK activation (Fig. 2a). Although PF-271 inhibited pY397 FAK, both vehicle and PF-271 groups demonstrated NF-κB phosphorylation at serine 536 (pS536 NF-κB) and rapid degradation of regulatory IκBα (Fig. 2a), indicating no change in NF-κB activation within 60 min of TNF-α stimulation. Dasatinib on the other hand, reduced pY418 Src and TNF-α-induced phosphorylation of JNK and p38 MAPKs, slightly reduced pS536 NF-κB but had no effect on ERK activation (Supp. Fig. 3a). Therefore, it is likely there are different patterns in MAPK regulation by PF-271 and Dasatinib, which may account for differential regulation of CAM expression in human ECs (Fig. 1a–d). Like PF-271 (Fig. 2a), FAK specific inhibitor PF-228 also reduced activation of ERK and JNK MAPKs but had no effect on NF-κB activation within 60 min of TNF-α treatment (Supp. Fig. 3b). To further test if FAK/Pyk2 activity regulates CAM expression through ERK and JNK MAPKs, we next treated HAoECs with either a MEK inhibitor (PD98059) or a JNK inhibitor (SP600125). Pretreatment with PD98059 only reduced TNF-α-induced expression of VCAM-1 (Supp. Fig. 4a). On the other hand, SP600125 treatment reduced expression of VCAM-1, ICAM-1, and E-selectin (Supp. Fig. 4b), suggesting that JNK signaling may have a larger affect than ERK on pro-inflammatory CAM expression. Additionally, knockdown of either FAK or Pyk2 reduced TNF-α-induced activation of ERK and JNK (Supp. Fig. 5). Compared to FAK/Pyk2 dual inhibition, MAPK inhibition was not as effective in reducing TNF-α-induced CAM expression, suggesting that regulation of pro-inflammatory genes is more complex and other signaling pathways may be involved.

### FAK/Pyk2 inhibition reduces TNF-α-induced inflammatory molecule transcription in human endothelial cells

Inhibition of MAPK signaling facilitates transcription of several pro-inflammatory genes. For instance, ERK inhibition reduces gene expression through reduced phosphorylation of Elk-1 transcription factor^46^. We hypothesized that PF-271 might inhibit TNF-α-induced transcription of VCAM-1, ICAM-1, and E-selectin potentially through blockade of MAPK associated signaling pathways. RT-qPCR data showed that FAK inhibition abrogated TNF-α-mediated transcription of these CAMs (Fig. 2b). However, FAK specific inhibition with PF-228 only reduced TNF-α-stimulated VCAM-1 transcript levels which matches with immunoblot results (Supp. Fig. 3c, Fig. 1c, and Supp. Fig. 1b). Since numerous pro-inflammatory genes are activated via the MAPK signaling pathways, we used an inflammatory RT-qPCR array for human cytokines, chemokines, and their receptors to determine which genes were regulated by FAK/Pyk2. Interestingly, PF-271 reduced TNF-α-induced expression of several genes known to promote vascular inflammatory diseases, such as CXCL1, CX3CR1, MCP-1, and IP-10 (Fig. 2d and Supp. Table 1)^47–55^. We verified our RT-qPCR screen by designing our own primers to investigate FAK/Pyk2 activity-mediated CXCL11, MCP-1, and IP-10 expression following TNF-α stimulation. Indeed, FAK/Pyk2 inhibition reduced TNF-α-induced transcription of CXCL11, MCP-1, and IP-10 in HAoECs (Fig. 2e). As FAK activity has previously been implicated to be important for MCP-1 expression in other cell types^56–58^, we also found decreased MCP-1 protein expression in HAoECs upon FAK/Pyk2 inhibition (Fig. 2f). These data demonstrate that FAK/Pyk2 activity is critical not only in promoting CAM expression, but also in the expression of many other pro-inflammatory genes through MAPK-mediated transcriptional activation.

### FAK/Pyk2 inhibition reduces TNF-α-induced monocyte adhesion and transmigration on endothelial cells

**Figure 3:**
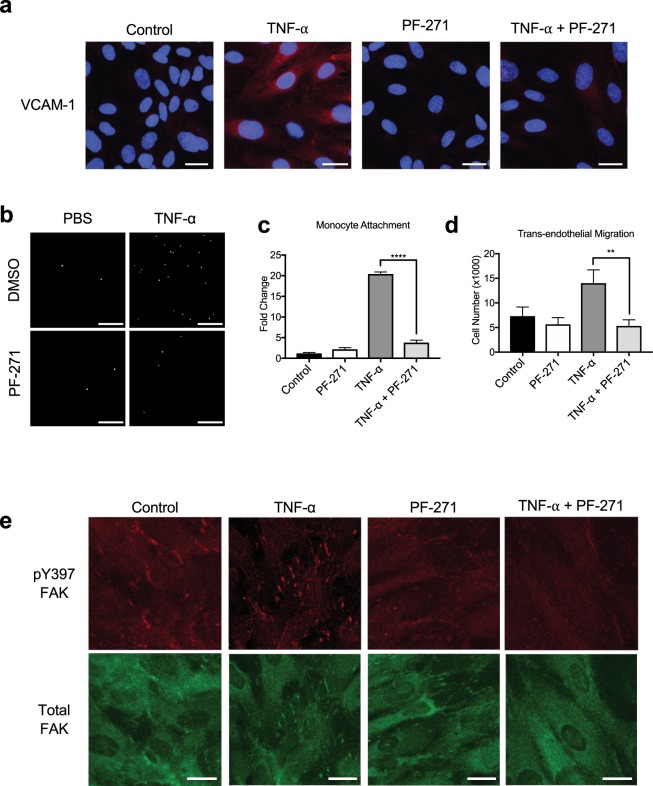
FAK/Pyk2 inhibition reduces TNF-α-induced *in vitro* inflammation. (**a**) HAoECs were treated with PF-271 (2.5 μM) for 1 h prior to TNF-α (10 ng/ml, 6 h) stimulation. Staining for VCAM-1 (red) and DAPI (blue) are shown. Scale bar (20 μm). (**b-c**) Monocyte adhesion assay. Primary mouse monocytes from bone marrow were labeled using Cell Tracker Green. HAoECs were treated with DMSO or PF-271 (2.5 μM) for 1 h prior to TNF-α (10 ng/ml, 6 h) stimulation. Monocytes were allowed to attach for 1 h prior to fixation. (**b**) Images of attached monocytes are shown. Scale bar (200 μm). (**c**) Attached monocytes were enumerated (n = 3, ±SEM). (**d**) HAoECs onto collagen I (10 μg/ml) coated Boyden chamber were treated with DMSO or PF-271 (2.5 μM) for 1 h prior to TNF-α (10 ng/ml, 6 h) stimulation. THP-1 cells were allowed to transmigrate the endothelial layer for 16 h. Transmigrated THP-1 cells were quantified (n = 3, ±SEM). (**e**) HAoECs were treated with PF-271 (2.5 μM) for 1 h prior to TNF-α (10 ng/ml, 6 h) stimulation. Staining of active FAK (pY397 FAK, red) or total FAK (green) are shown. Scale bar (20 μm). **p < 0.01, ****p < 0.0001.

Since expression of VCAM-1, ICAM-1, and E-selectin on the surface of ECs plays a crucial role in the recruitment of monocytes to sites of inflammation, we next investigated if FAK/Pyk2 inhibition can reduce monocyte recruitment during inflammation *in vitro*. In line with our immunoblotting and RT-qPCR results (Figs 1a and 2b), we observed increased VCAM-1 staining in TNF-α treated HAoECs, which was blocked by PF-271 treatment (Fig. 3a). In monocyte attachment assay, TNF-α increased attachment of primary mouse monocytes to HAoECs, and FAK/Pyk2 inhibition reduced monocyte attachment by approximately 10-fold compared to TNF-α alone (Fig. 3b,c). As trans-endothelial migration is another key step in the recruitment of leukocytes to a site of inflammation^59^, we examined the ability of PF-271 to inhibit monocyte transmigration across ECs using a Boyden chamber. PF-271 reduced transmigration by 60% compared to TNF-α-stimulated group (Fig. 3d). Interestingly, TNF-α treated HAoECs show increased localization of FAK in focal adhesions (Fig. 3e), which correlates with increased FAK activity confirmed by pY397 FAK staining (Fig. 3e). Importantly, PF-271 reduces FAK localization to focal adhesions and FAK activation upon TNF-α stimulation (Fig. 3e). Taken together, these data suggest that dual FAK/Pyk2 inhibition reduces TNF-α-mediated vascular inflammation *in vitro*, supporting the idea that FAK/Pyk2 activity is critical to promote vascular inflammation.

### FAK, Pyk2, and Src differentially regulate IL-1β-mediated inflammatory gene expression

**Figure 4:**
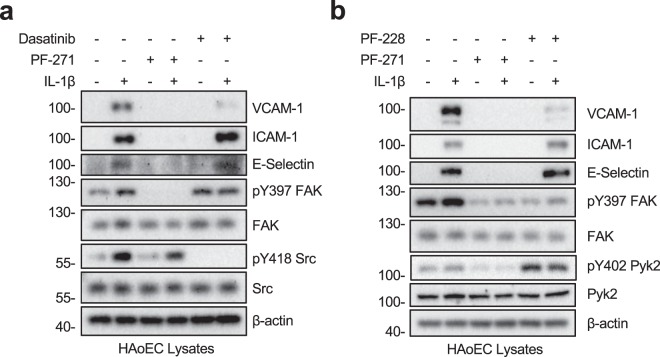
FAK/Pyk2 inhibition blocks IL-1β-mediated pro-inflammatory adhesion molecule expression in HAoECs. (**a**) HAoECs were treated with DMSO, a dual FAK/Pyk2 inhibitor (PF-271, 2.5 μM) or a Src inhibitor (Dasatinib, 1 μM) for 1 h prior to IL-1β (20 ng/ml, 6 h) stimulation. Cropped images of immunoblotting for VCAM-1, ICAM-1, E-selectin, active FAK (pY397 FAK), FAK, active Src (pY418 Src), Src, and β-actin as loading control are shown. Full length blots shown in Supplemental Fig. 11. (**b**) HAoECs were treated with DMSO, a dual FAK/Pyk2 inhibitor (PF-271, 2.5 μM) or a FAK inhibitor (PF-228, 10 μM) for 1 h prior to IL-1β (20 ng/ml, 6 h) stimulation. Cropped images of immunoblotting for VCAM-1, ICAM-1, E-selectin, pY397 FAK, FAK, pY402 Pyk2, Pyk2, and β-actin as loading control are shown. Full length blots shown in Supplemental Fig. 12.

Since TNF-α signaling shares many of the downstream pathways as IL-1β, we have attempted to evaluate if PTK activity is important in IL-1β-mediated pro-inflammatory gene expression. Similar to what we saw with TNF-α (Fig. 1a), PF-271 reduced expression of VCAM-1, ICAM-1, and E-selectin following IL-1β stimulation (Fig. 4a). Although IL-1β strongly increased activation of Src (pY418), Src inhibition with Dasatinib only reduced IL-1β-stimulated expression of VCAM-1 (Fig. 4a). These results are consistent with our observation upon TNF-α treatment (Fig. 1a), suggesting that both TNF and IL receptors utilize a similar set of PTKs to promote inflammatory gene expression in human ECs.

FAK specific inhibition with PF-228 reduced expression of VCAM-1 but did not alter expression of ICAM-1 and E-selectin (Fig. 4b). As seen in TNF-α signaling, FAK specific inhibition with PF-228 increased active pY402 Pyk2 (Fig. 4b). While Pyk2 activity alone is sufficient to mediate IL-1β-mediated ICAM-1 and E-selectin expression, VCAM-1 expression requires FAK activity. In agreement with reduction in pro-inflammatory CAMs, PF-271 also reduced attachment of primary mouse monocytes to IL-1β stimulated HAoECs (Supp. Fig. 6a,b). These data suggest that FAK and Pyk2 cooperatively mediate TNF-α and IL-1β pathways, representing multi-layer regulatory mechanisms that control gene expression of CAMs during vascular inflammation.

### FAK/Pyk2 inhibition reduces IL-1β-induced inflammatory molecule transcription through MAPKs in human endothelial cells

**Figure 5:**
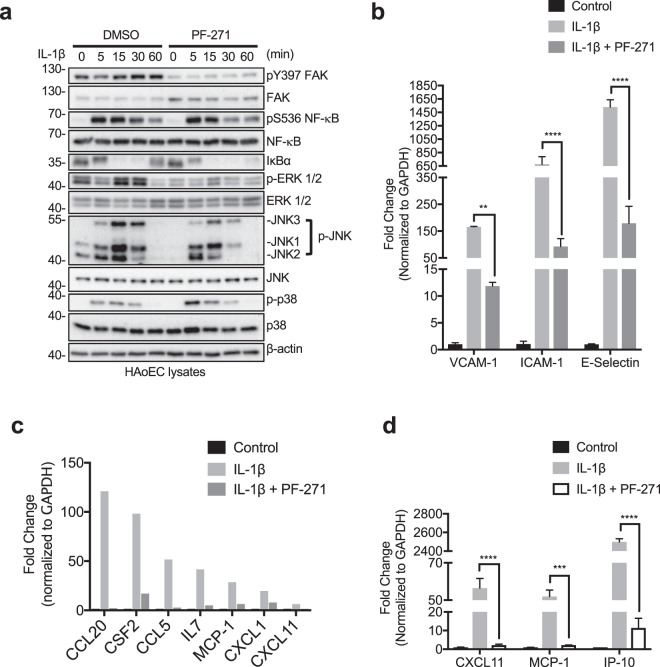
FAK/Pyk2 activity promotes IL-1β-mediated pro-inflammatory molecule expression via transcriptional control. (**a**) HAoECs were treated with DMSO or PF-271 (2.5 μM) for 1 h prior to IL-1β (20 ng/ml) stimulation for 0 to 60 min. Cropped images of immunoblotting for pY397 FAK, FAK, active NF-κB (pS536 NF-κB), NF-κB, IκBα, active ERK (p-ERK), ERK, active JNK (p-JNK1, 2, and 3), JNK, active p38 (p-p38), p38, and β-actin as loading control are shown. Full length blots shown in Supplemental Fig. 13. (**b**) HAoECs were treated with DMSO or PF-271 (2.5 μM) for 1 h prior to IL-1β (20 ng/ml, 6 h) stimulation. RNA was collected, and RT-qPCR for cell adhesion molecules was performed (n = 3, ±SEM). (**c**) HAoECs were treated with DMSO or PF-271 (2.5 μM) for 1 h prior to IL-1β (20 ng/ml, 24 h) stimulation. RNA was collected, and RT-qPCR using an array for inflammatory cytokines, chemokines and their receptors. Shown are a selection of genes that are important in vascular inflammation and were reduced by PF-271 treatment upon IL-1β stimulation. (**d**) CXCL11, MCP-1, and IP-10 mRNA levels were verified by RT-qPCR (n = 3, ±SEM). **p < 0.01, ***p < 0.001, ****p < 0.0001.

As MAPK and NF-κB signaling are important in IL-1β-mediated inflammatory molecule expression, we evaluated the effectiveness of FAK/Pyk2 inhibition on MAPK and NF-κB activation. Similar to TNF-α (Fig. 2a), PF-271 also reduced IL-1β-mediated activation of both ERK and JNK but slightly increased p38 MAPK (Fig. 5a). In agreement with reduced MAPK activation, treatment with PF-271 also reduced transcription of VCAM-1, ICAM-1, and E-selectin compared to IL-1β alone (Fig. 5b). Using an inflammatory RT-qPCR array, we identified several genes that were downregulated in PF-271 treated cells (Fig. 5c and Supp. Table 2). We further verified that FAK/Pyk2 inhibition reduced IL-1β-mediated transcription of MCP-1, CXCL11, and IP-10 (Fig. 5d), similar to what was observed with TNF-α (Fig. 2e). These findings suggest that FAK/Pyk2 function as key PTKs downstream of both TNF-α and IL-1β receptors in human ECs.

### FAK/Pyk2 inhibition reduces VCAM-1 expression and macrophage recruitment in *ApoE*−/− mice upon high fat diet

**Figure 6:**
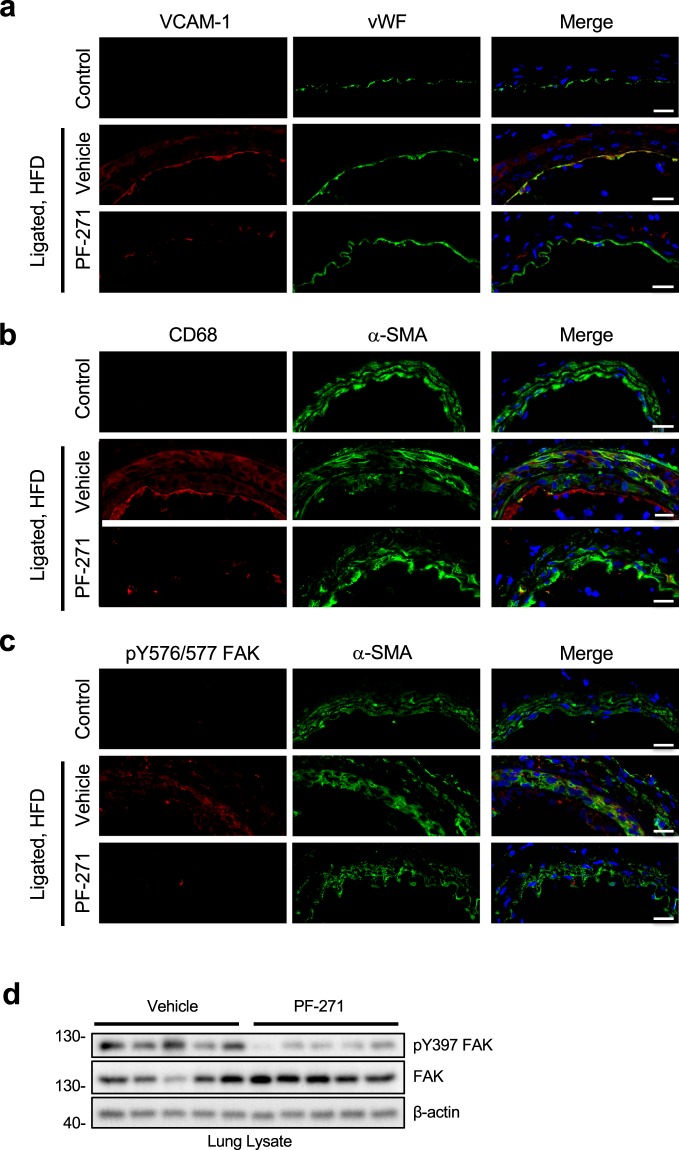
FAK/Pyk2 activity controls vascular inflammation *in vivo* mouse carotid ligation model. Ligated carotid arteries from 8-week-old *ApoE*−/− mice were harvested after fed a high fat diet (HFD) for 1 week with or without PF-271. Normal arteries from *ApoE*−/− mice fed with normal chow were used as control. (**a**) Sections were stained with VCAM-1 (red), vWF (green), and DAPI (blue). Scale bar (20 μm). (**b**) Macrophage infiltration was monitored with anti-CD68 (red) staining. Anti-α-SMA (green) and DAPI (blue) were used as counterstain. Scale bar (20 μm). (**c**) FAK activity was evaluated by staining anti-pY576/577 FAK (red). Anti-α-SMA (green) and DAPI (blue) were used as counterstain. Scale bar (20 μm). (**d**) Cropped images of immunoblotting of lung lysates of active FAK (pY397 FAK), total FAK, and β-actin as loading control are shown. Full length blots shown in Supplemental Fig. 14.

*ApoE*−/− mice are an excellent model system for vascular inflammation since mice will experience vascular inflammation after initiation of a high fat diet (HFD)^20,60^. To accelerate the induction of vascular inflammation, we performed carotid artery ligation in *ApoE*−/− mice^61^. Carotid ligation led to expression of VCAM-1 within the vessel when compared to a control artery (no ligation or HFD) (Fig. 6a). Strong VCAM-1 expression was detected on ECs as seen by co-localization with vWF (Fig. 6a). Importantly, PF-271 treatment (35 mg/kg, twice daily by oral gavage, for 2 weeks) significantly reduced ligation-induced VCAM-1 expression (Fig. 6a). Since VCAM-1 plays a key role in leukocyte recruitment^62^, we determined whether PF-271 reduced leukocyte recruitment by analyzing the artery sections with a macrophage marker, anti-CD68. While vehicle group showed an increase in macrophage recruitment around layers of EC, SMC, and adventitia compared to control artery, PF-271 efficiently blocked macrophage recruitment in the area (Fig. 6b). Carotid artery ligation and HFD increased FAK activity, verified by pY576/577 FAK staining, while control artery showed very little FAK activity (Fig. 6c). Additionally, we have confirmed efficacy of PF-271 in mice by performing pY397 FAK immunoblotting with lung lysates and demonstrated that PF-271 significantly reduces FAK activity *in vivo* (Fig. 6d). Therefore, we have concluded that vascular insults increase FAK activity, which plays a key role in promoting vascular inflammation by inducing VCAM-1 expression and promoting macrophage infiltration. Based on our findings, FAK inhibitors could be used as a therapeutic target for treating vascular inflammatory diseases.

## Discussion

During vascular inflammation, TNF-α and IL-1β create a local site of inflammation by promoting inflammatory CAM expression and leukocyte recruitment to the endothelium. Based on studies using pan-tyrosine kinase inhibitors, it has long been speculated that PTK activity is important for TNF-α and IL-1β signaling. Here, we are the first study that distinguishes the contributions of individual PTKs in these signaling pathways through the use of small molecule inhibitors that inhibit FAK/Pyk2 (PF-271, VS-6063) and Src family kinases (Dasatinib). While Src inhibition reduces VCAM-1, FAK/Pyk2 inhibition significantly blunted the expression of three important CAMs: VCAM-1, ICAM-1, and E-selectin (Figs. 1a, 4a and Supp. Fig. 1e). This finding clearly showed that activity of individual PTK families differentially regulate pro-inflammatory molecule expression in human ECs. While the FAK specific inhibitor PF-228 efficiently blocked VCAM-1 expression, it increased Pyk2 activity (Figs. 1e and 4b), this result suggests that Pyk2 activity may contribute to the expression of ICAM-1 and E-selectin. Since there is no Pyk2 specific inhibitor available, to test CAM expression in a Pyk2-specific manner we performed FAK or Pyk2 knockdown using siRNA. Interestingly, both siFAK and siPyk2 were able to reduce CAM expression to a similar degree (Fig. 1f), suggesting that expression of both FAK and Pyk2 is important for TNF-α-induced CAM expression in ECs. Together, these data suggest that dual inhibition of FAK/Pyk2 is required for reducing inflammatory CAM expression.

Previously, we and others reported that FAK activity regulates VCAM-1 expression during development and through different vascular insults such as TNF-α, IL-1β, and oxidized LDL^41,63^. Our current study identified that FAK/Pyk2 activity was also important for TNF-α and IL-1β-stimulated transcription of ICAM-1 and E-selectin (Figs. 2b and 5b). We also identified several cytokines and chemokines, such as MCP-1, IP-10, and CXCL11, that were significantly downregulated by FAK/Pyk2 inhibition in HAoECs following TNF-α or IL-1β stimulation (Figs. 2d and 5c). MCP-1 and IP-10 have been shown to play key roles in vascular inflammatory diseases like atherosclerosis by reducing recruitment of monocytes^53^ and T-cells^55^, respectively. Although CXCL11 was one of our strongest candidates identified from the cytokine array, it is currently unknown what role, if any, CXCL11 plays during vascular inflammation. While it has been shown that activation of CXCR7, the receptor for CXCL11, using a synthetic ligand was able to reduce atherosclerosis in mice^64^, it is not known if CXCL11 binding to CXCR7 will result in the same response. Overall, these results show that FAK/Pyk2 inhibition could prove beneficial in reducing inflammation.

While proximal signaling of TNF and IL receptors are quite different, their downstream intracellular pathways share common molecular events such as activation of MAPKs and the canonical NF-κB pathway to induce inflammatory gene expression^26,27^. This study identified distinct MAPK signaling pathways affected by FAK/Pyk2, FAK, or Src inhibition. While PF-271 and PF-228 reduced ERK and JNK MAPKs (Fig. 2a and Supp. Fig. 3b), Dasatinib did not affect ERK MAPK activation (Supp. Fig. 3a). It seems there are different patterns in MAPK regulation by PF-271, PF-228, and Dasatinib, which may result in distinct regulation of CAM expression. Although Src inhibition slightly reduced activation of NF-κB immediately following TNF-α stimulation, it failed to reduce expression of ICAM-1 and E-selectin, indicating Src-mediated NF-κB signaling is not the only mechanism responsible for induction of CAM expression during vascular inflammation. While PF-271 dramatically reduced transcription of CAMs, it had no effect on TNF-α-induced activation of NF-κB within 60 min TNF-α or IL-1β stimulation (Figs. 2a and 5a,b). Together, these data suggested that FAK/Pyk2 activity is critical not only in promoting CAM expression, but also in the expression of many pro-inflammatory genes via MAPK-mediated transcriptional activation. Additionally, while treatment with a MEK inhibitor (PD98059) was only able to reduce VCAM-1 expression (Supp. Fig. 4a), a JNK inhibitor (SP600125) reduced expression of all three inflammatory CAMs (Supp. Fig. 4b). These data suggest that ERK and JNK may activate different transcriptional factors responsible for selective CAM expression. Intriguingly, while IL-4 activates FAK, FAK expression was not required for VCAM-1 expression, and expression of the endogenous FAK inhibitor FRNK (FAK related non-kinase) blocked IL-4-induced VCAM-1 expression^65^. These findings suggested that FAK involvement in inflammation signaling may be far more complicated.

Our study found that FAK/Pyk2 inhibition reduces expression of CAMs both *in vitro* and *in vivo*, which is a key step in leukocyte extravasation^59^. Our data strongly suggest that FAK inhibitors could be used as a therapeutic target for reducing leukocyte infiltration. Previous studies have shown that FAK is important for disrupting endothelial barrier function^66^, and that FAK inhibition might prevent the extravasation of cells from the blood to the surrounding tissue. Interestingly, treatment with diperoxovanadate, which activates tyrosine kinases and inhibits tyrosine phosphatases, decreased EC barrier stability and non-selective inhibition of PTKs with genistein improved barrier function^67^. Increased phosphorylation of FAK and focal adhesions were also observed prior to decreased EC barrier function^67^. While TNF-α decreases endothelial permeability both *in vitro* and *in vivo*^68–70^, and we observed increased active FAK at focal adhesions and increased monocyte trans-endothelial migration, it is not known how FAK activity regulates endothelial junction integrity upon cytokine stimulation.

In summary, we have concluded that vascular insults increase FAK activity which plays a key role in promoting vascular inflammation by inducing expression of important CAMs and promoting macrophage infiltration. Therefore, FAK/Pyk2 inhibitors could be useful as an anti-inflammatory drug in the treatment of vascular inflammatory diseases.

## Materials and Methods

### Cell culture and reagents

Human aortic endothelial cells (HAoECs) were purchased from LifeLine Cell Technologies and maintained in VascuLife VEGF media (LifeLine Cell Technolgies). Cells between passages 3 and 11 were used for experiments. HAoECs were pretreated for 1 h with either the FAK/Pyk2 inhibitor PF-271 (MedKoo), the FAK inhibitor PF-228 (MedKoo), the FAK/Pyk2 inhibitor PF-271 VS-6063 (MedChemExpress), the Src inhibitor Dasatinib (Selleckchem), the MEK inhibitor PD98059 (Tocris), or the JNK inhibitor SP600125 (Selleckchem) prior to stimulation with either TNF-α (R&D) or IL-1β (Miltenyi Biotec). THP-1 cells were purchased from ATCC and maintained in RPMI Medium (Sigma-Aldrich) supplemented with 10% FBS (Omega Scientific). Collagen I from rat tail was purchased from BD Biosciences.

### siRNA transfection

HAoECs were transfected with FAK, Pyk2, or a non-targeting siRNA for 36 h using Lipofectamine RNAi MAX according to the manufacturer’s instructions (Life Technologies) prior to TNF-α stimulation. The siRNA sequences used were: siFAK-1^71^ 5′-UGUUGGUUUAAAGCGAUUUtt-3′, siFAK-2 5′-GAUGUUGGUUUAAAGCGAUtt-3′, siPyk2-1^71^ 5′-CGUCAUCUUCACGGACAGAtt-3′, siPyk2-2^71^ 5′-UAUCCUCAAGGUCUGCUUCtt-3′, and non-targeting siRNA 5′-UAACGACGCGACGACGUAAtt-3′.

### Antibodies

Full list of antibodies used in this study can be found in Supplemental Table 3.

### Primary mouse monocyte isolation

Primary mouse monocytes were isolated from bone marrow of wild-type C57BL/6 male mice using the Monocyte Isolation Kit for mice according to manufacturer’s instructions (Miltenyi Biotec). Primary mouse monocytes were incubated with CellTracker Green CMFDA Dye for 30 min according to manufacturer’s instructions (Life Technologies) and used for monocyte attachment assay.

### Monocyte attachment assay

Confluent HAoECs were pre-treated with DMSO or PF-271 for 1 h prior to treatment with TNF-α or IL-1β for 6 h. HAoECs were washed 3 times with PBS at RT and stained primary mouse monocytes were allowed to adhere for 1 h. Unbound cells were washed away using PBS, and adhered cells were fixed using 4% PFA for 10 min at RT. Adhered cells were visualized using fluorescent microscopy and enumerated.

### Trans-endothelial migration assay

HAoECs were seeded onto Boyden chamber (8 um pore size, Millipore) coated with collagen type I from rat tail. After reaching confluency, HAoECs were pre-treated with DMSO or PF-271 for 1 h prior to treatment with TNF-α for 6 h. 1 × 10^6^ THP-1 cells were then added and allowed to migrate through the EC layer for 16 h. Number of THP-1 cells that migrated through the chamber were enumerated.

### RT-qPCR array

Total RNA was collected using NucleoSpin RNA kit (Macherey-Nagel). cDNA was prepared using the SuperScript III Reverse Transcriptase kit according to manufacturer’s instructions (Invitrogen). Real time qPCR was performed using the Power SYBR Master Mix (Applied Biosystems). PCR reaction was carried out on the CFX Connect Real-Time PCR Detection System (Bio-Rad). Cycling parameters were: 50 °C for 2 min, 95 °C for 2 min, then 40 cycles of 95 °C for 15 sec and 60 °C for 1 min. Data were calculated by ΔΔCt using β-actin or GAPDH as an internal control and fold change was determined by comparison to vehicle-treated controls for each sample. The Human Inflammatory Cytokines and Receptors RT^2^ Profiler PCR Array (Cat. No. 330231 PAHS-011ZA; QIAGEN) was used to evaluate expression of 84 genes. Primers used for RT-qPCR can be found in Supplemental Table 4.

### Elisa

HAoECs were treated with a FAK inhibitor for 1 h prior to TNF-α stimulation. Conditioned media was collected, and MCP-1 protein expression was determined using human MCP-1 ELISA kit according to manufacturer’s instructions (Affymetrix). Expression of CAMs from cell lysates was determined using ELISAs against human VCAM-1 (Raybiotech), human ICAM-1 (Raybiotech), or human E-selectin (Abcam) according to manufacturer’s instructions.

### Immunocytochemistry

HAoECs were seeded onto gelatin-coated coverslips in a 24-well plate and allowed to reach confluency. HAoECs were pretreated with either DMSO or the FAK/Pyk2 inhibitor PF-271 for 1 h prior to stimulation with TNF-α. Then cells were fixed using 2% PFA in PBS for 20 min at room temperature (RT) or with 100% ice cold methanol for 10 min at −20 °C and washed with PBS. Cells were blocked and permeabilized in a blocking buffer (10% goat serum, 0.3% Triton X-100, 1x PBS) for 45 min at RT. Slides were incubated overnight at 4 °C with primary antibody in dilution buffer (1% goat serum, 1% BSA, 0.3% Triton X-100, 0.01% sodium azide, 1xPBS). Coverslips were washed with PBS and then incubated with Alexa Fluor secondary antibodies in the dark for 1 hr at RT. Slides were washed with PBS and nuclei were stained using DAPI for 2 min. Coverslips were mounted onto slides using Fluoromount-G (SouthernBiotech) and held in place using clear nail polish. Fluorescent microscopy was performed using a Nikon A1 Confocal Microscope.

### Carotid artery ligation model in *ApoE*−/− mice

Animal experiments were approved by and performed in accordance with the guidelines of the University of South Alabama Institutional Animal Care and Use Committee (Protocol# 1007032). Complete ligation of left carotid artery was performed as previously described^61^. *ApoE*−/− mice were purchased from Jackson Laboratory. Male mice (6–8 weeks of age) were anesthetized with ketamine/xylazine administered intraperitoneally (IP). Hair from throat area was removed and the area was sterilized using Betadine. A small 2 cm incision was made parallel to the trachea to allow access to the left carotid artery. The left carotid was ligated using sterile 6-0 silk suture proximal to the left carotid branch, and the incision was stitched closed. Following morning, mice started a high fat/high cholesterol diet (21% fat, 0. 2% cholesterol, Tekladd) and treated twice daily with either vehicle (30% 2-Hydroxypropyl-β-cyclodextrin, 2.5% Dextrose) or PF-271 (35 mg/kg, twice daily) by oral gavage for 1 week. Previous studies have shown that dosages up to 50 mg/kg are not toxic in mice, and that treatment twice a day had the largest effect on suppressing FAK activity^72,73^.

### Collection of tissue samples

Mice were euthanized via IP injection of ketamine/xylazine. Mice were perfused with 5 mM EDTA in PBS through the left ventricle. Next, mice were perfused with PBS, followed by 4% PFA in PBS to fix tissue structure. Prior to fixation, one lobe of the lung was isolated and lysed in 1 mL of RIPA buffer (1% Triton X-100, 50 mM HEPES pH 7.4, 150 mM NaCl, 10% glycerol, 1.5 mM MgCl_2_, 10 mM sodium pyrophosphate, 0.1% SDS, protease inhibitor (Roche)). Lungs were sonicated, diluted 1:5 in fresh RIPA buffer, and sonicated again. Lysates were incubated with 50 μl of Sephadex beads on rotator for 10 min at 4 °C. Lung lysates were centrifuged at 20,000 g for 2 h at 4 °C, and supernatant was mixed with 4x SDS Protein Sample Buffer and boiled for 5 min. Samples were then run on SDS-PAGE to evaluate active FAK (pY397 FAK) levels.

### Immunohistochemistry

After fixation, both the carotid arteries were separated from the surrounding tissue, embedded in optimal cutting temperature (O.C.T., Fisher Scientific) and frozen at −80 °C. Frozen blocks were allowed to reach −20 °C in Cryostat prior to sectioning. Samples were cut at a thickness of 7 μm and were allowed to adhere to RT glass slides. Frozen sections were allowed to dry at RT for 10 min, and then fixed with 4% PFA in PBS for 10 min at RT. Slides were then washed in PBS and then blocked using ICC blocking buffer (1% BSA, 1% goat serum, 0.3% Trion X-100, 0.01% sodium azide, 1x PBS) for 1 h at RT. Sections were then incubated with Mouse on Mouse solution according to manufacturer’s instructions (Vector Labs). Sections were incubated overnight with primary antibodies in PBS at 4 °C. Samples were washed with PBS for 15 min at RT and incubated with secondary antibodies for 1 h in the dark at RT. Samples were washed with PBS and nuclei were stained with DAPI for 2 min at RT. Cover slides were mounted using Fluoromount-G (SouthernBiotech) to protect fluorescent staining and held in place with clear nail polish. Fluorescent microscopy was performed using Nikon A1 Confocal microscope.

### Statistical analysis

Statistical significance was evaluated by ANOVA, and p < 0.05 was considered to be statistically significant. Analyses were performed using GraphPad Prism.

## Supplementary information

Supplemental information
