# Supplementary material for: FAK and Pyk2 activity promote TNF-α and IL-1β-mediated pro-inflammatory gene expression and vascular inflammation
Source: Sci Rep. 2019 May 20;9:7617. doi: 10.1038/s41598-019-44098-2 (PMC6527705; doi:10.1038/s41598-019-44098-2)
Supplement: Supplementary file 1 — Supplemental information [file 41598_2019_44098_MOESM1_ESM.pdf]

Supplemental information

FAK and Pyk2 activity promote TNF-α and IL-1β-mediated pro-inflammatory gene expression and vascular inflammation

James M. Murphy[1], Kyuho Jeong[1], Yelitza A. R. Rodriguez[1], Jung-Hyun Kim[2], Eun-Young Erin Ahn[2], and Ssang-Taek Steve Lim[1,3]

[1] Department of Biochemistry and Molecular Biology, College of Medicine, University of South Alabama, Mobile, AL 36688

[2] Mitchell Cancer Institute, University of South Alabama, Mobile, AL 36604

Supplemental figure legends

Supplemental Fig. 1. Dual FAK/Pyk2 pharmacological inhibitors block VCAM-1 expression in HAoECs. (a) HAoECs were treated for DMSO or a dual FAK/Pyk2 inhibitor (PF-271, 1-10 µM) for 1 h prior to TNF-α (10 ng/ml, 6 h) stimulation. Cropped images of immunoblots of VCAM-1, active FAK (pY397 FAK), FAK, and β-actin as loading control are shown. Full length blots shown in Supplemental Figure 15. **(b)** Expression levels of VCAM-1, ICAM-1, and E-selectin were quantified using Image J (n=3, ±SEM). **(c)** HAoECs were treated with DMSO or a FAK inhibitor (PF-228, 1-10 µM) for 1 h prior to TNF-α (10 ng/ml, 6 h) stimulation. Croppped images of immunoblots of VCAM-1, active FAK (pY397 FAK), FAK, and β-actin as loading control are shown. Dotted line shows were membrane was cut to remove unnecessary lanes. Full length blots shown in Supplemental Figure 16. **(d)** Expression levels of VCAM-1, ICAM-1, and E-selectin were quantified using Image J (n=3, ±SEM). **(e)** HAoECs were treated for with DMSO or a dual FAK/Pyk2 inhibitor (VS-6063, 1-10 µM) for 1 h prior to TNF-α (10 ng/ml, 6 h) stimulation. Cropped images of immunoblots of VCAM-1, ICAM-1, E-selectin, active FAK (pY397 FAK), FAK, and β-actin as loading control are shown. Full length blots shown in Supplemental Figure 17.

Supplemental Fig 2. FAK or Pyk2 knockdown reduces TNF-α-induced pro-inflammatory adhesion molecule expression in HAoECs. HAoECs were transfected with either siControl (400 pmol, 36 h) and either **(a)** 2 different FAK siRNA (400 pmol, 36 h) or **(b)** 2 different Pyk2 siRNA (400 pmol, 36 h) prior to TNF-α (10 ng/ml, 4 h) stimulation. Cropped images of immunoblotting for VCAM-1, ICAM-1, E-selectin, FAK, Pyk2, and β-actin as loading control are shown. Full length blots shown in **(a)** Supplemental Figure 18 and **(b)** Supplemental Figure 19.

Supplemental Fig. 3. Pharmacological Src and FAK inhibition reduce MAPK signaling pathway activation. (a) HAoECs were treated with DMSO or a Src inhibitor (Dasatinib, 1 μM) for 1 h prior to stimulation with TNF-α (10 ng/ml) for 0 to 60 min. Cropped images of immunoblotting for active FAK (pY397 FAK), FAK, active Src (pY418 Src), Src, active NF-κB (pS536 NF-κB), NF-κB, active ERK (p-ERK), ERK, active JNK (p-JNK), JNK, active p38 (p-p38), p38, and β-actin as loading control are shown. Full length blots shown in Supplemental Figure 20. **(b)** HAoECs were treated with DMSO or a FAK inhibitor (PF-228, 10 μM) for 1 h prior to stimulation with TNF-α (10 ng/ml) for 0 to 60 min. Cropped Images of immunoblotting for active FAK (pY397 FAK), FAK, active NF-κB (pS536 NF-κB), NF-κB, active ERK (p-ERK), ERK, active JNK (p-JNK), JNK, active p38 (p-p38), p38, and β-actin are shown. Full length blots shown in Supplemental Figure 21. **(c)** HAoECs were treated with DMSO or PF-228 (10 μM) for 1 h prior to TNF-α (10 ng/ml, 6 h) stimulation. RNA was collected and RT-qPCR was performed (n= 3, ±SEM). * $p<0.05$.

Supplemental Fig 4. MAPK pathway inhibition reduces TNF-α-induced pro-inflammatory adhesion molecule expression in HAoECs. (a) HAoECs were treated with DMSO or a MEK inhibitor (PD98059, 30 μM) for 1 h prior to TNF-α (10 ng/ml, 4 h) stimulation. Cropped images of immunoblotting for VCAM-1, ICAM-1, E-selectin, p-ERK, and β-actin as loading control are shown. Full length blots shown in Supplemental Figure 22. **(b)** HAoECs were treated with DMSO or a JNK inhibitor (SP600125, 25 μM)

for 1 h prior to TNF-α (10 ng/ml, 4 h) stimulation. Cropped images of immunoblotting for VCAM-1, ICAM-1, E-selectin, p-JNK, and β-actin as loading control are shown. Full length blots shown in Supplemental Figure 23.

Supplemental Fig. 5. FAK or Pyk2 knockdown reduces TNF-α-induced activation of ERK and JNK in HAoEcs. HAoECs were transfected with either siControl, siFAK-2, or siPyk2-2 siRNA (400 pmol, 36 h) prior to TNF-α (10 ng/ml, 15 min) stimulation. Cropped images of immunoblotting for FAK, Pyk2, p-ERK, p-JNK, and GAPDH as loading control are shown. Full length blots shown in Supplemental Figure 24.

Supplemental Fig. 6. FAK/Pyk2 inhibition reduces IL-1β-mediated monocyte attachment. (a-b) Primary mouse monocytes were isolated from bone marrow and labeled using Cell Tracker Green. HAoECs were treated with DMSO or a dual FAK/Pyk2 inhibitor (PF-271, 2.5 µM) for 1 h prior to IL-1β (20 ng/ml, 6 h) stimulation. Scale bar (200 µm). **(a)** Images of attached monocytes are shown. **(b)** Attached monocytes were enumerated (n=3, ±SEM). **** $p<0.0001$

Supplemental Fig. 7. Shown are full length blots of cropped images (red boxes) shown in Figure 1a of the text. Identification of bands was based on predicted molecular weight.

Supplemental Fig. 8. Shown are full length blots of cropped images (red boxes) shown in Figure 1b of the text. Identification of bands was based on predicted molecular weight.

Supplemental Fig. 9. Shown are full length blots of cropped images (red boxes) shown in Figure 1d of the text. Identification of bands was based on predicted molecular weight.

Supplemental Fig. 10. Shown are full length blots of cropped images (red boxes) shown in Figure 2a of the text. Identification of bands was based on predicted molecular weight.

Supplemental Fig. 11. Shown are full length blots of cropped images (red boxes) shown in Figure 4a of the text. Identification of bands was based on predicted molecular weight.

Supplemental Fig. 12. Shown are full length blots of cropped images (red boxes) shown in Figure 4b of the text. Identification of bands was based on predicted molecular weight.

Supplemental Fig. 13. Shown are full length blots of cropped images (red boxes) shown in Figure 5a of the text. Identification of bands was based on predicted molecular weight.

Supplemental Fig. 14. Shown are full length blots of cropped images (red boxes) shown in Figure 6d of the text. Identification of bands was based on predicted molecular weight.

Supplemental Fig. 15. Shown are full length blots of cropped images (red boxes) shown in Supplemental Figure 1a. Identification of bands was based on predicted molecular weight.

Supplemental Fig. 16. Shown are full length blots of cropped images (red boxes) shown in Supplemental Figure 1c. Identification of bands was based on predicted molecular weight.

Supplemental Fig. 17. Shown are full length blots of cropped images (red boxes) shown in Supplemental Figure 1e. Identification of bands was based on predicted molecular weight.

Supplemental Fig. 18. Shown are full length blots of cropped images (red boxes) shown in Supplemental Figure 2a. Identification of bands was based on predicted molecular weight.

Supplemental Fig. 19. Shown are full length blots of cropped images (red boxes) shown in Supplemental Figure 2b. Identification of bands was based on predicted molecular weight.

Supplemental Fig. 20. Shown are full length blots of cropped images (red boxes) shown in Supplemental Figure 3a. Identification of bands was based on predicted molecular weight.

Supplemental Fig. 21. Shown are full length blots of cropped images (red boxes) shown in Supplemental Figure 3b. Identification of bands was based on predicted molecular weight.

Supplemental Fig. 22. Shown are full length blots of cropped images (red boxes) shown in Supplemental Figure 4a. Identification of bands was based on predicted molecular weight.

Supplemental Fig. 23. Shown are full length blots of cropped images (red boxes) shown in Supplemental Figure 4b. Identification of bands was based on predicted molecular weight.

Supplemental Fig. 24. Shown are full length blots of cropped images (red boxes) shown in Supplemental Figure 5. Identification of bands was based on predicted molecular weight.

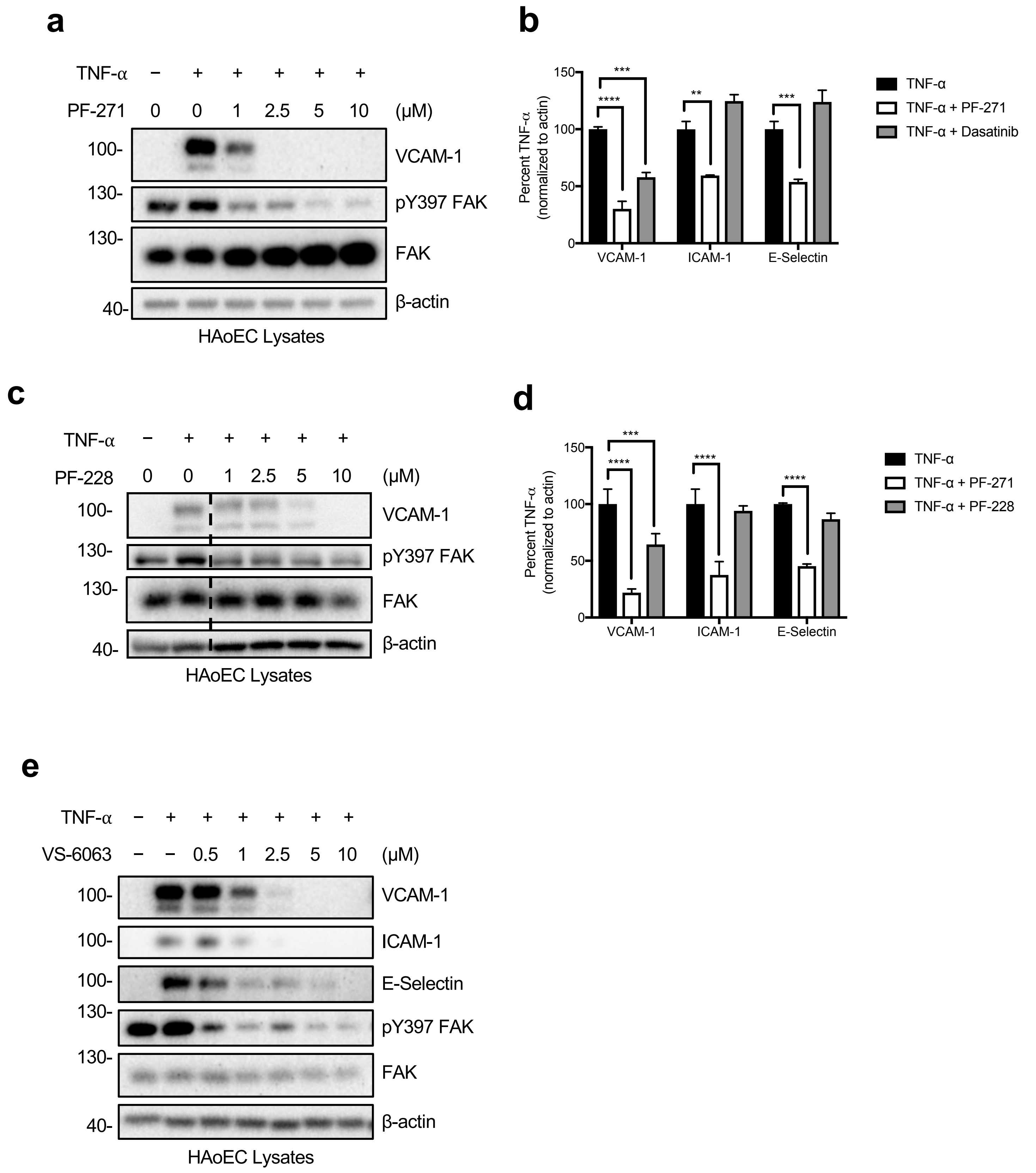

Supplemental Figure 1

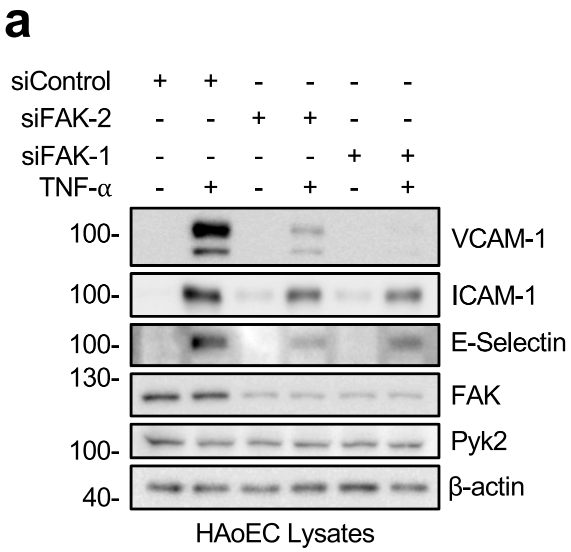

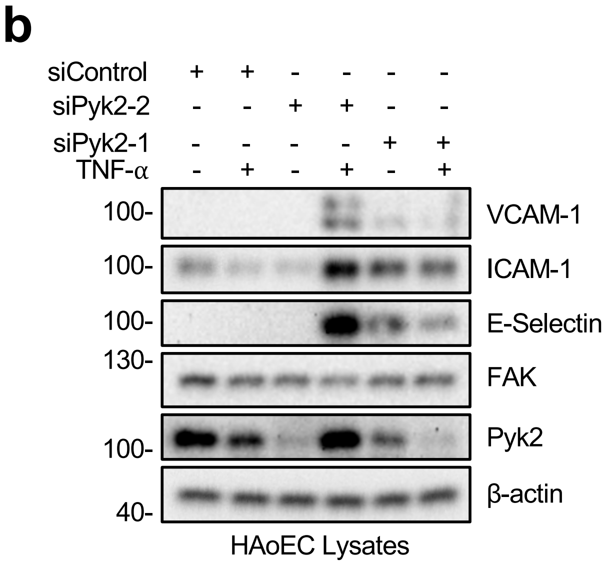

Supplemental Figure 2

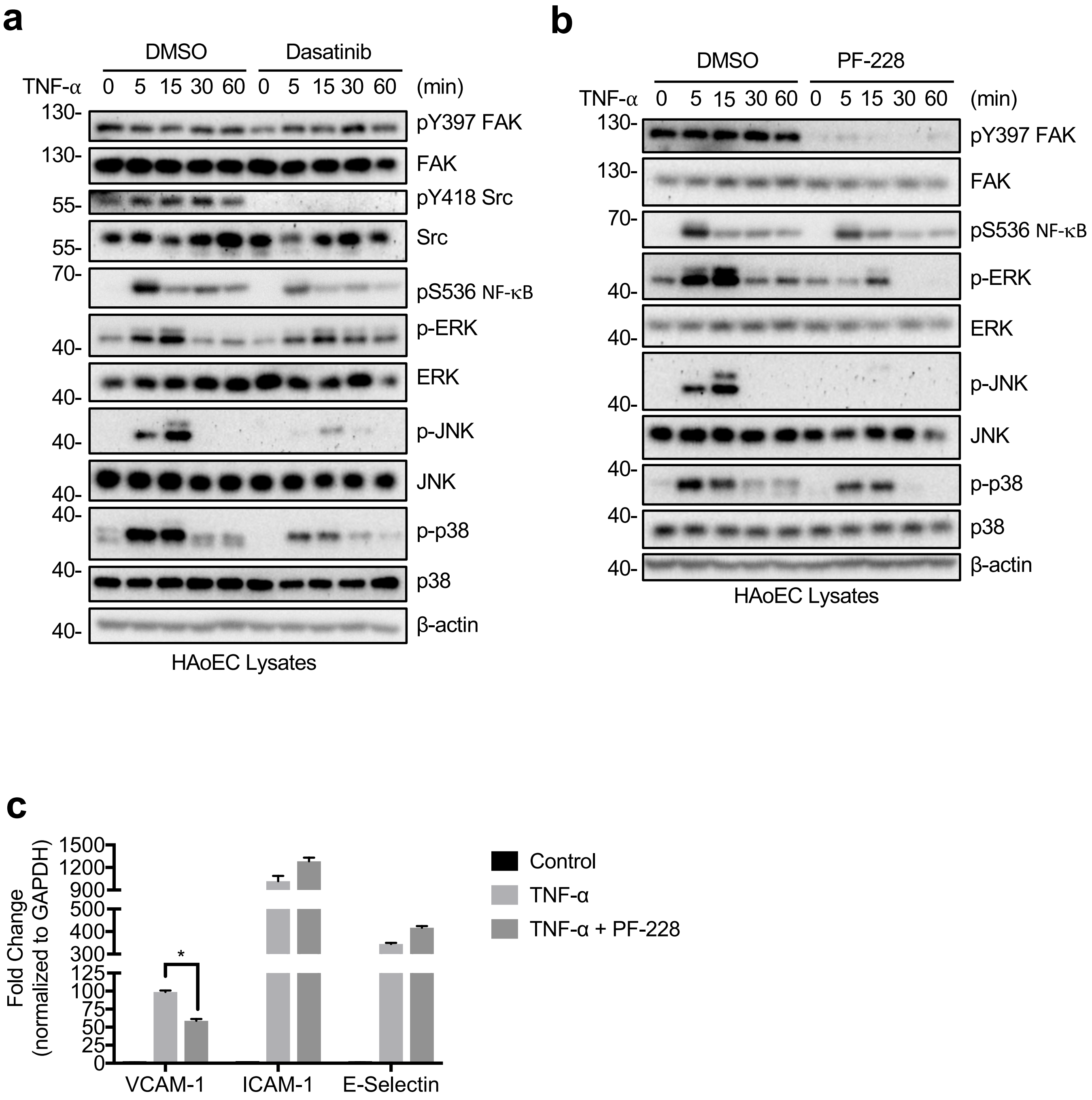

Supplemental Figure 3

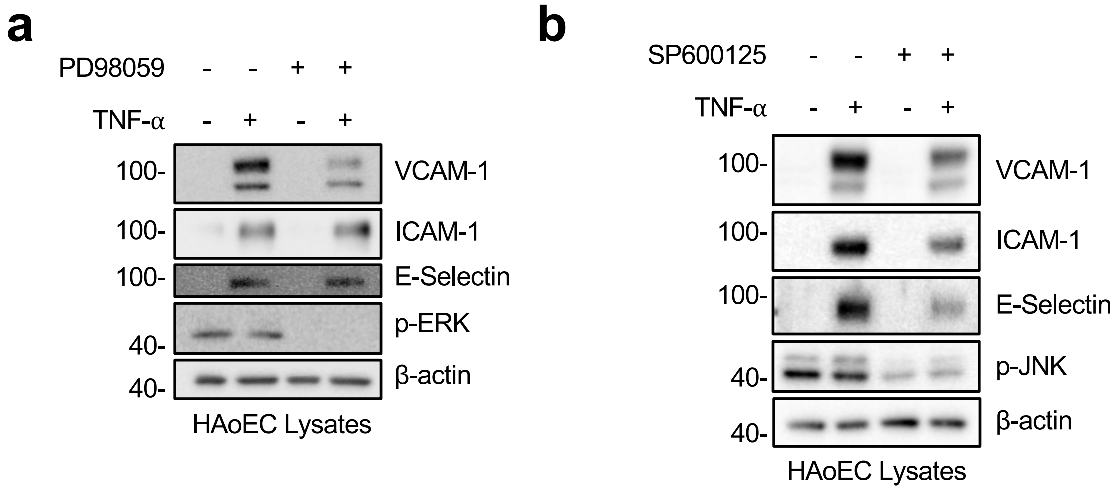

Supplemental Figure 4

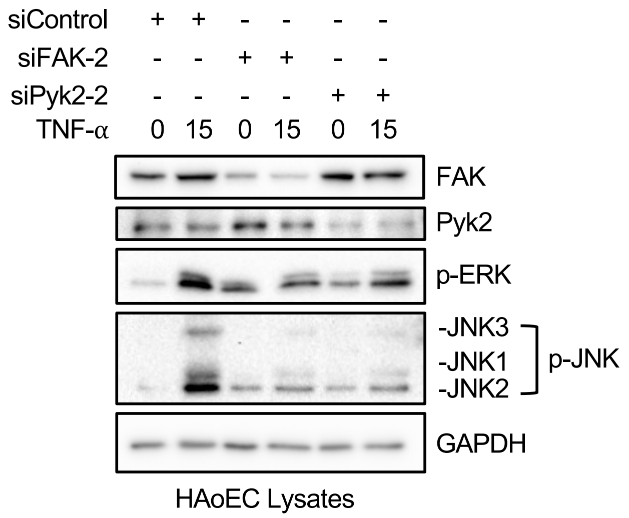

Supplemental Figure 5

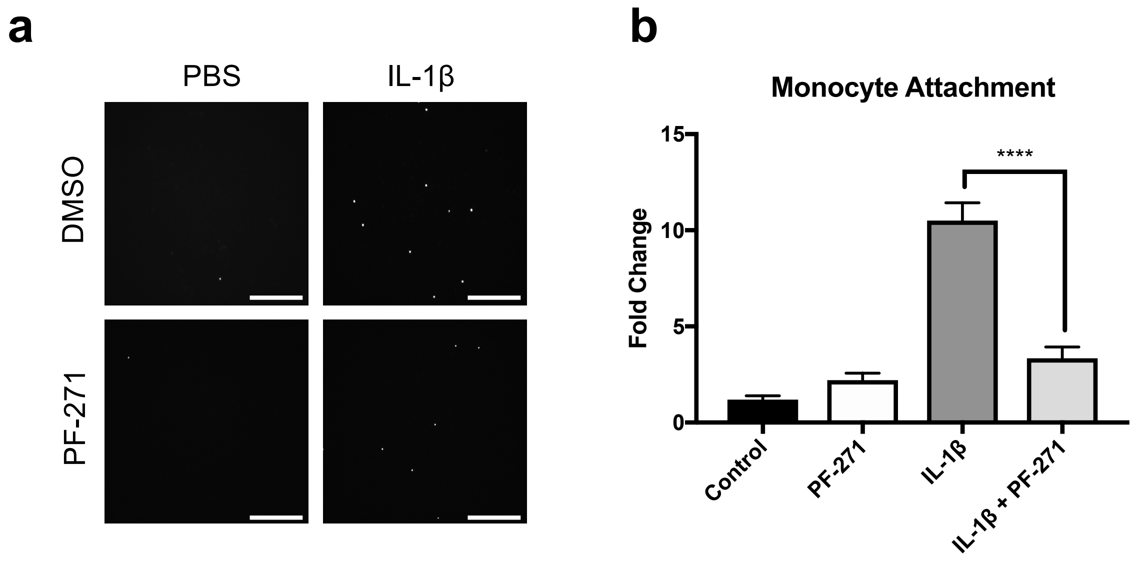

Supplemental Figure 6

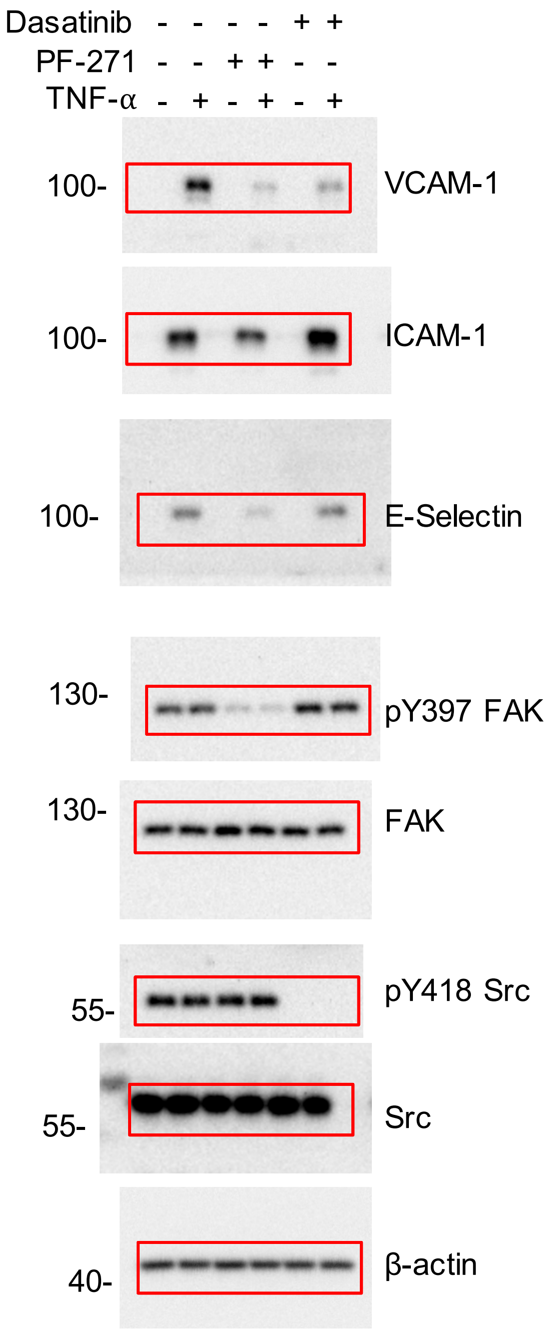

Supplemental Figure 7

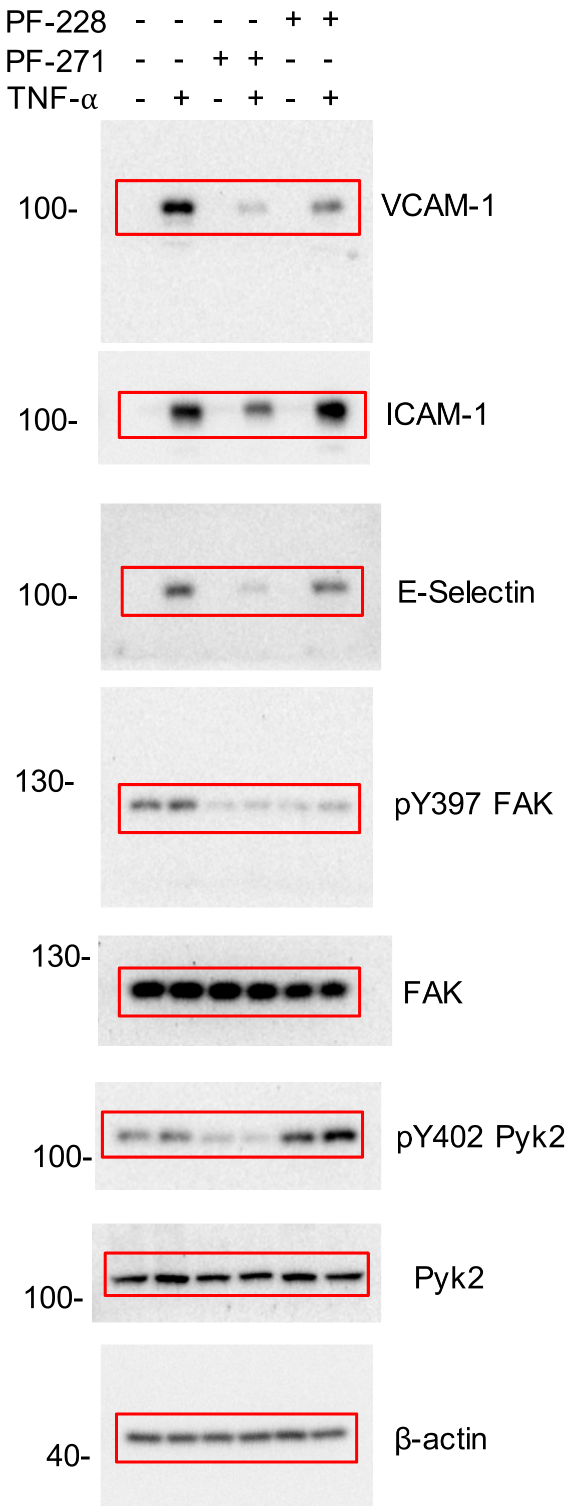

Supplemental Figure 8

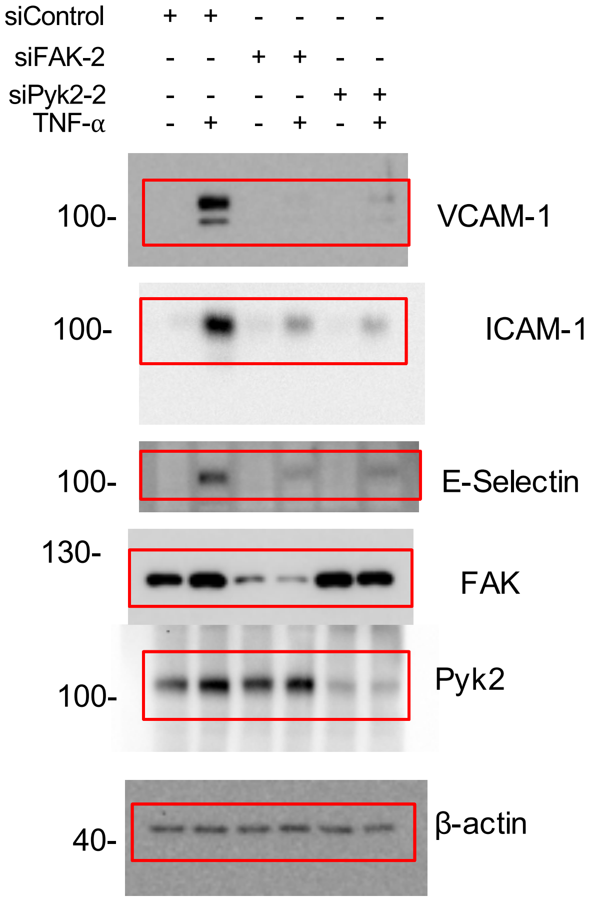

Supplemental Figure 9

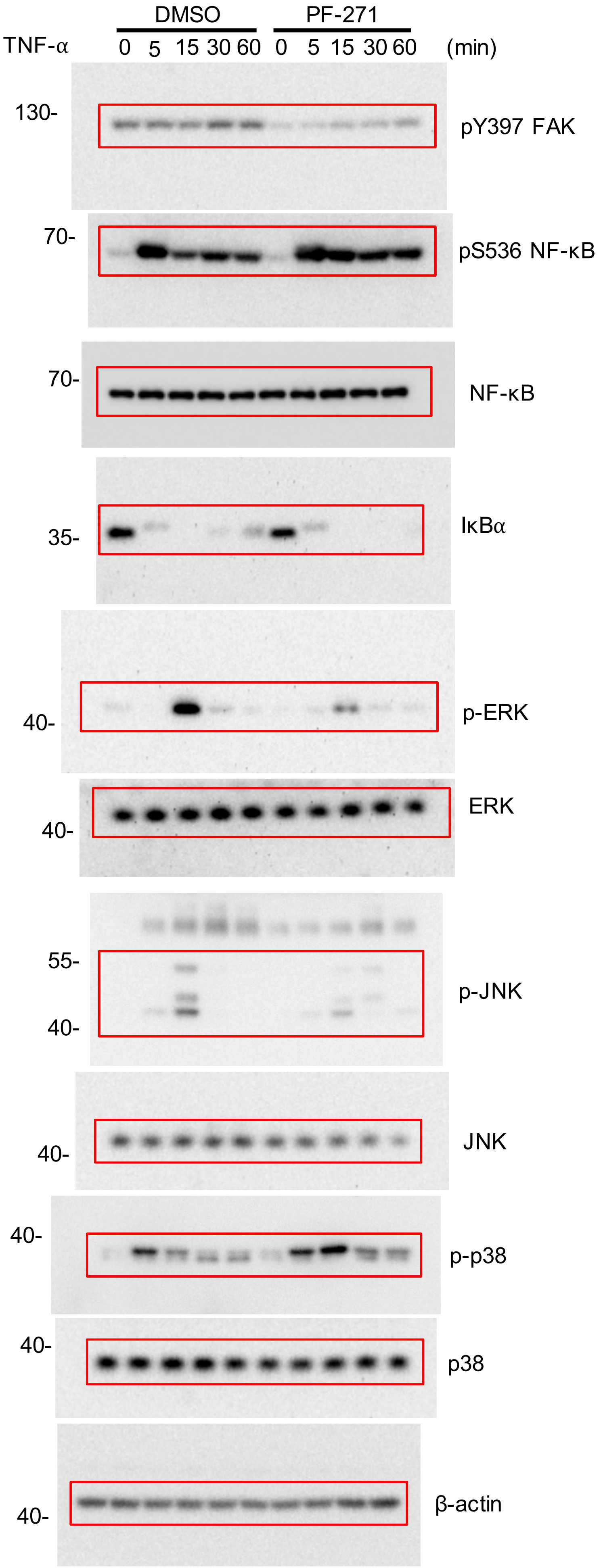

Supplemental Figure 10

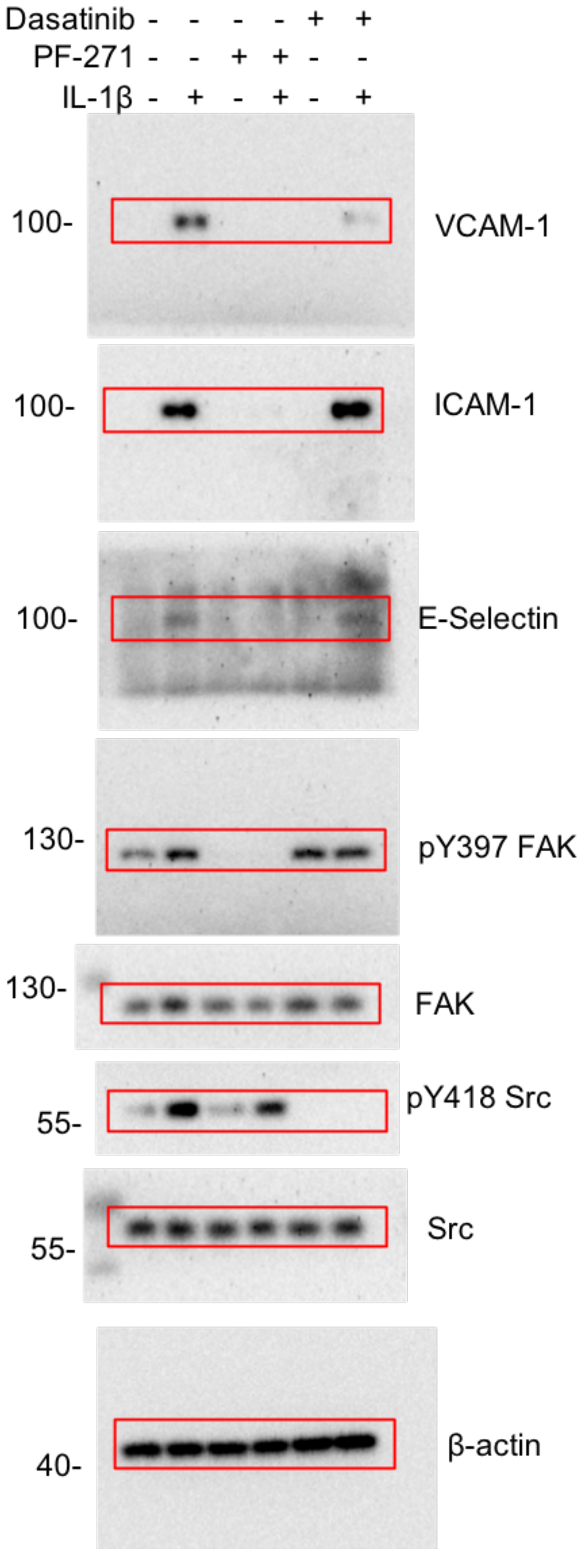

Supplemental Figure 11

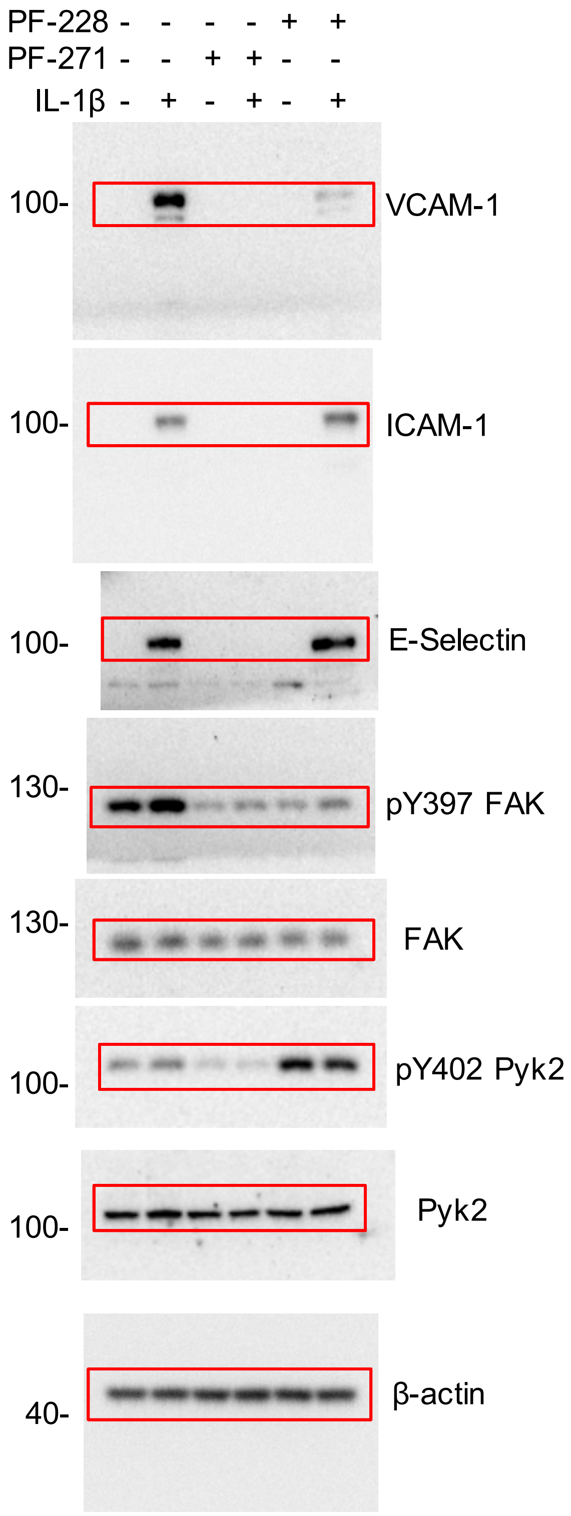

Supplemental Figure 12

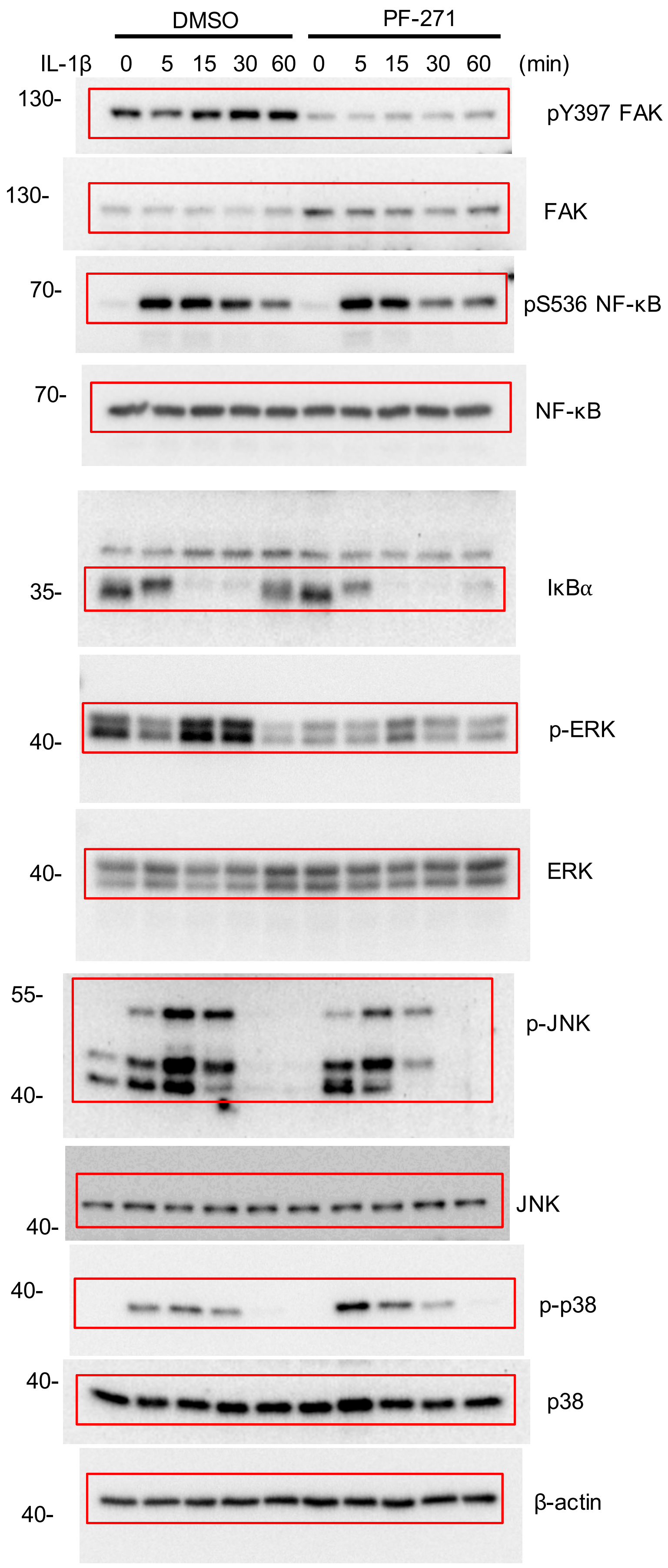

Supplemental Figure 13

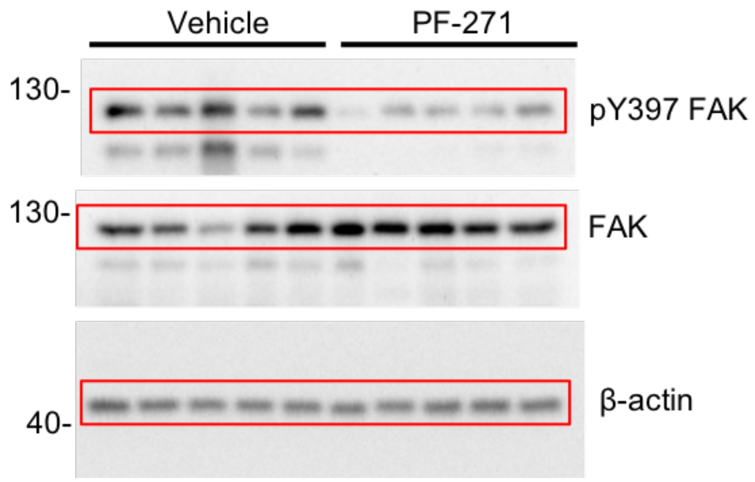

Supplemental Figure 14

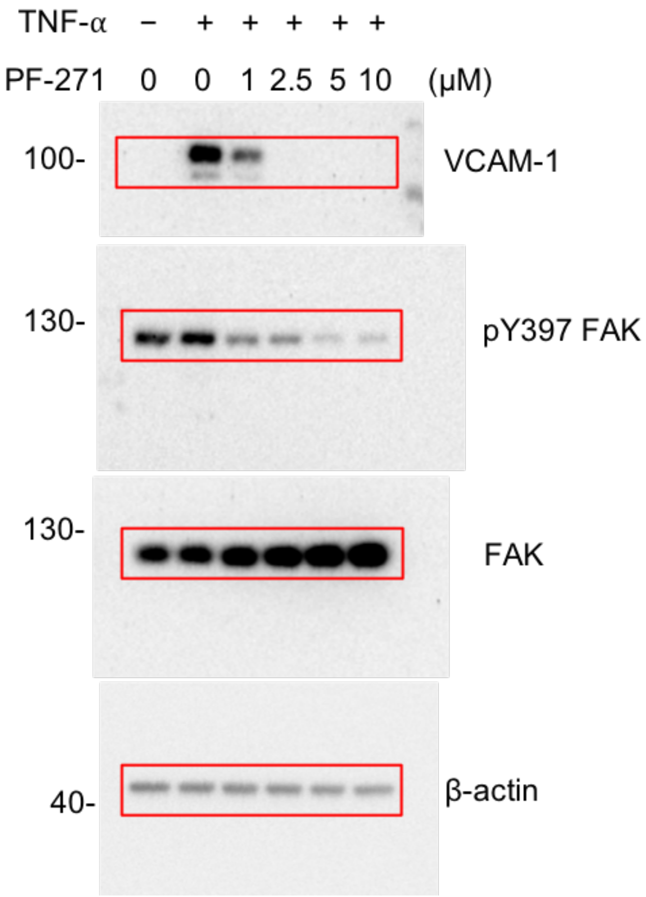

Supplemental Figure 15

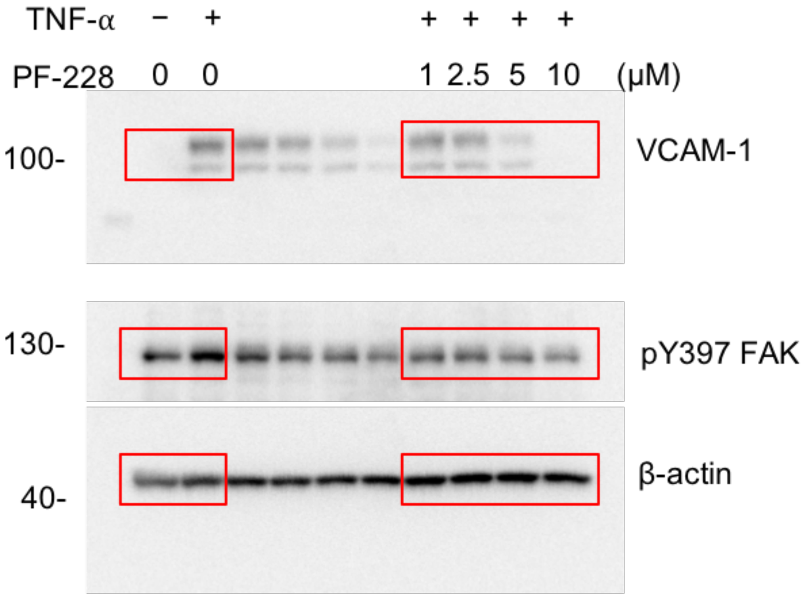

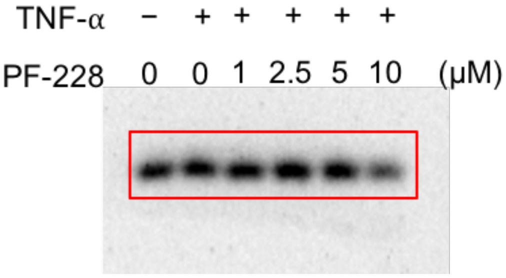

Supplemental Figure 16

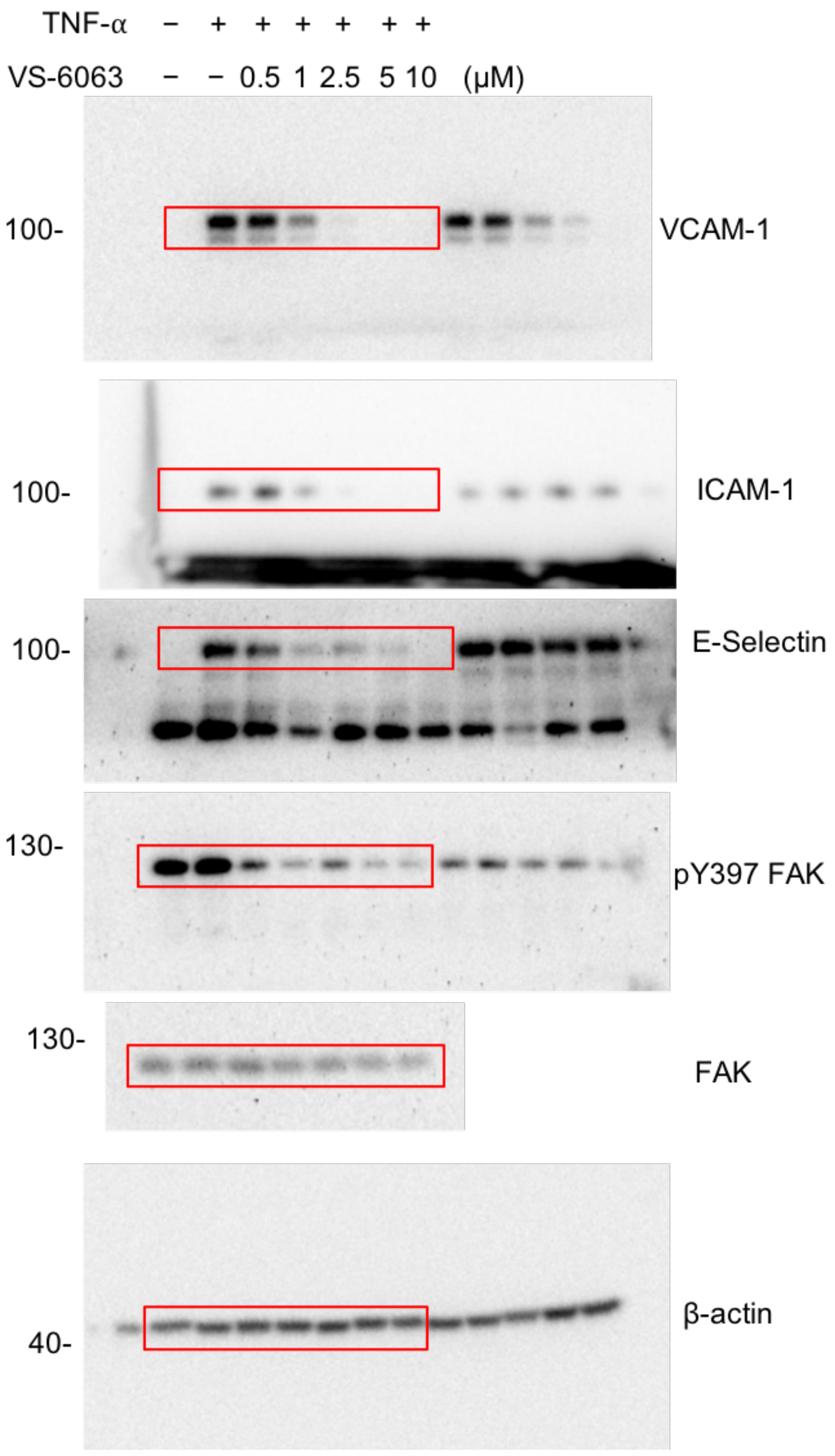

Supplemental Figure 17

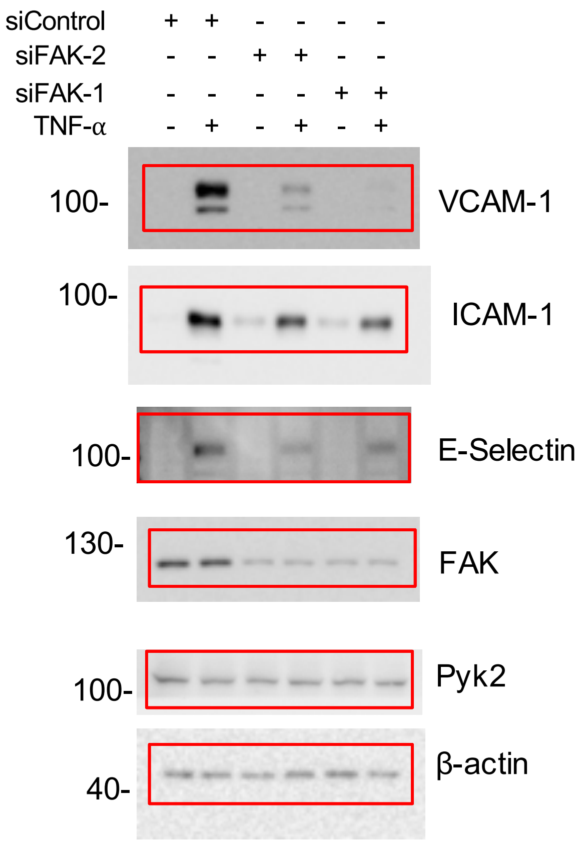

Supplemental Figure 18

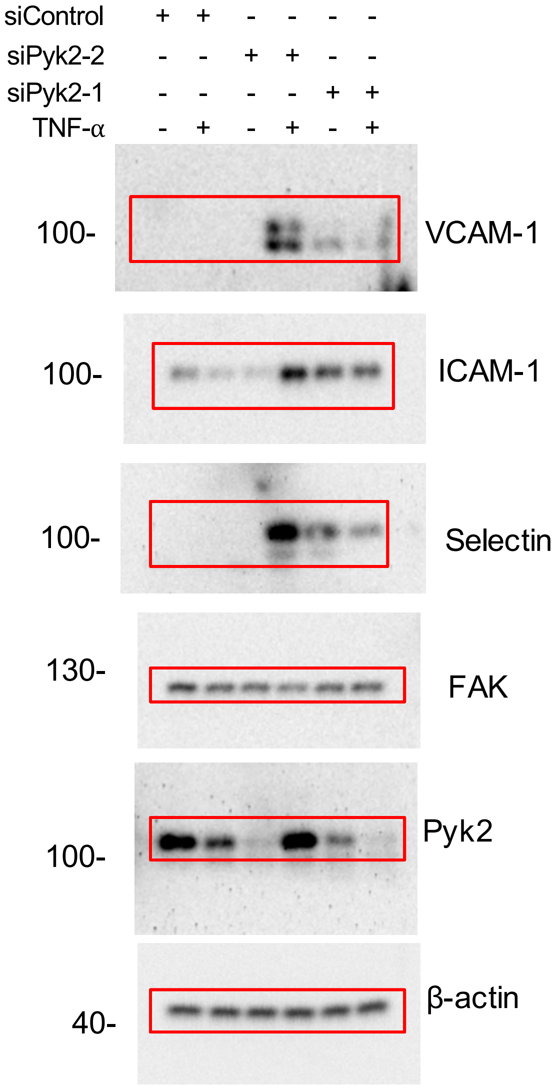

Supplemental Figure 19

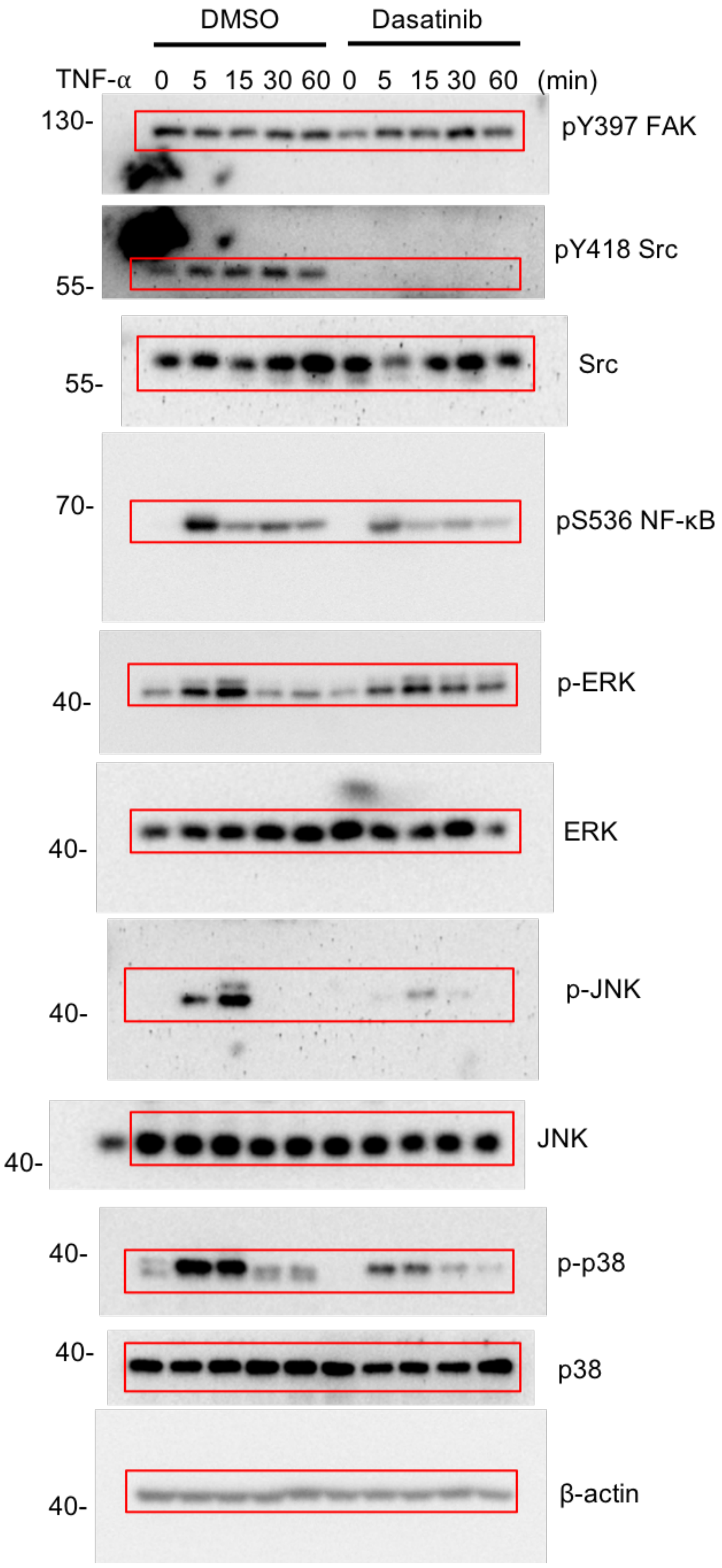

Supplemental Figure 20

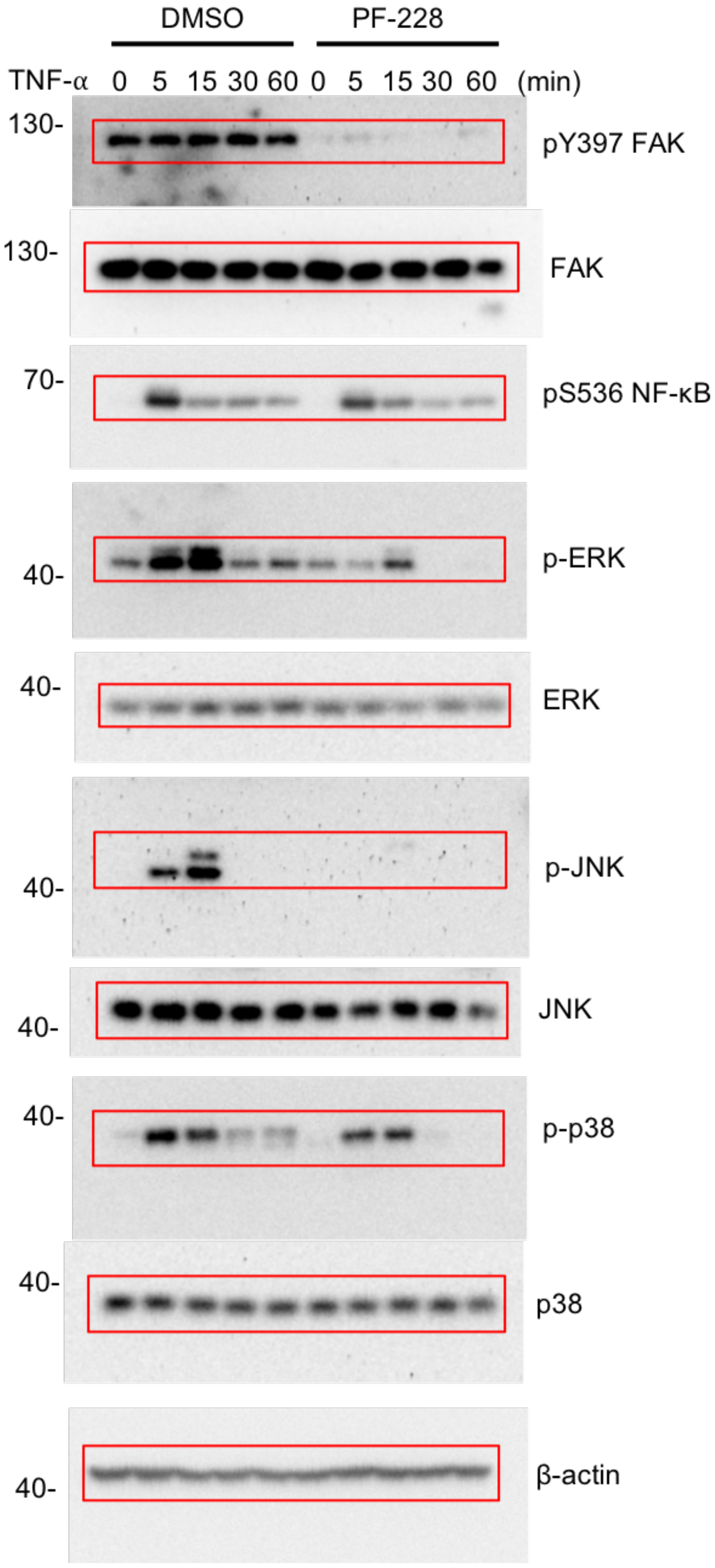

Supplemental Figure 21

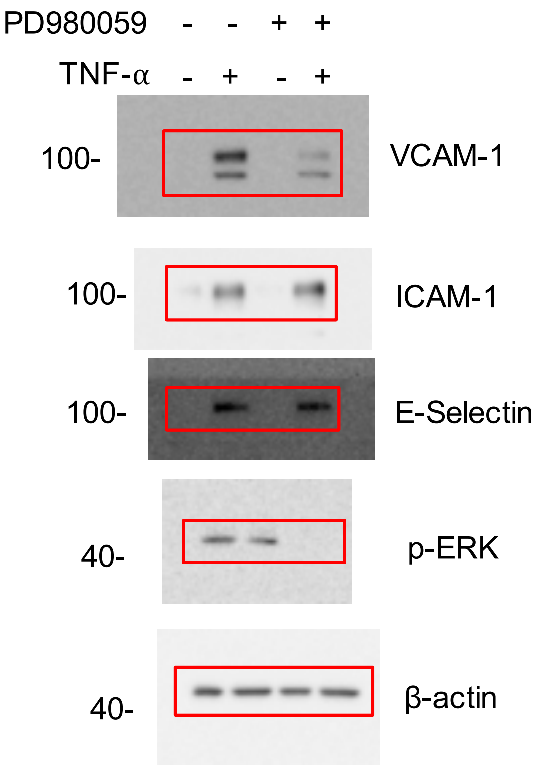

Supplemental Figure 22

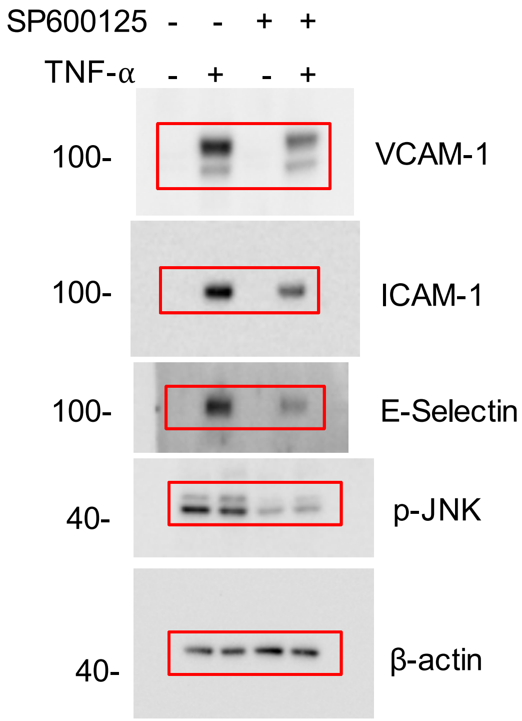

Supplemental Figure 23

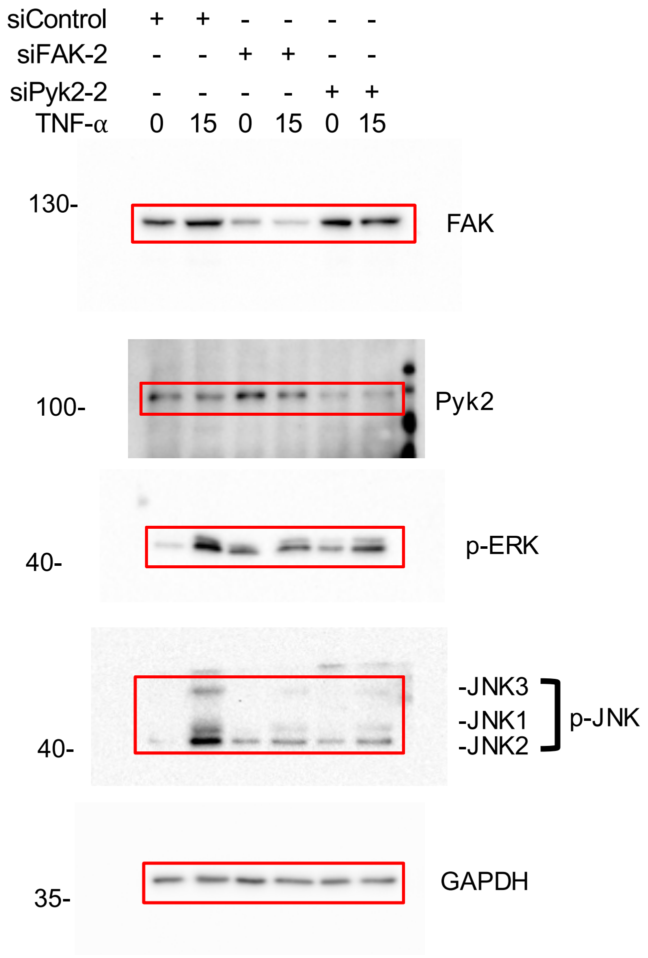

Supplemental Figure 24

Supplemental Table 1. Shown are fold changes above untreatead controls normalized to β-actin. mRNA levels were determined using the Human Inflammatory Cytokines and Receptors RT2 Profiler PCR Array (Cat. No. 330231 PAHS-011ZA; QIAGEN). Note: N/A: Not Available, no mRNA was detected

Gene Name	TNF-α	TNF-α + PF-271	Potential Role in Atherosclerosis	References
AIMP1	0.66	0.59		
BMP2	1.91	2.81		
C5	0.30	0.33		
CCL1	0.48	0.55		
CCL11	0.97	0.36		
CCL13	0.20	0.13		
CCL15	4.08	0.80		
CCL16	0.85	N/A		
CCL17	19.29	12.91	Upregulatead in human atherosclerotic plaques	[1]
MCP-1	47.84	27.28	Found in both mouse and human atherosclerosis. Knockout reduced atherosclerosis in *Ldlr-/-* mice	[2-4]
CCL20	41.36	9.51	Elevated levels found in hypercholesterolemic patients,	[5]
CCL22	N/A	N/A		
CCL23	0.59	0.10		
CCL24	1.51	0.09		
CCL26	0.43	0.39		
CCL3	1.38	0.04	Expressed in human atherosclerosis	[6]
CCL4	0.88	0.10	Expressed in human atherosclerosis	[6]
CCL5	1686.71	25.46	Expressed in both mouse and human atherosclerosis	[7,8]
CCL7	1.27	0.37		
CCL8	0.31	0.10		
CCR1	1.68	1.55	Knockout increased atherosclerosis in mice	[9]
CCR2	2.03	1.30	Knockout reduced atherosclerosis in mice	[10,11]
CCR3	0.51	0.10		
CCR4	30.48	1.97		
CCR5	2.75	0.26	Knockout reduced atherosclerosis in mice	[9,12]
CCR6	0.27	0.12	Knockout reduced atherosclerosis in mice	[13]
CCR8	0.57	0.15		
CD40LG	0.50	0.75		
CSF1	8.46	6.11	Knockout reduced atherosclerosis in mice. Reduced inflammation and increased macropahge apoptosis in lesions	[14,15]
CSF2	28.44	6.63	Knockout reduced atherosclerosis in mice	[16]
CSF3	21.71	0.26		
CX3CL1	81.01	18.77	Knockout reduced atherosclerosis in mice	[17]
CX3CR1	15.14	0.04	Knockout reduced atherosclerosis in mice	[18,19]
CXCL1	9.99	2.53	Increased in serum of patients with coronary artery diesease. Knockout reduced atherosclerosis in mice	[20,21]
IP-10	1992.00	7.21	Expressed in human atherosclerosis. Knockout reduced atherosclerosis in mice	[22,23
CXCL11	1675.06	26.35		
CXCL12	0.20	0.14	Found in human atherosclerosis, and reduced serum levels in coronary artery disease patients and old *ApoE-/-* mice	[24-26]
CXCL13	0.88	0.45		
CXCL2	9.32	2.41		
CXCL3	25.99	8.51		
CXCL5	3.32	1.88		
CXCL6	11.79	0.96		
CXCL9	45.25	0.31		
CXCR1	0.71	1.48		
CXCR2	0.38	0.44	Increased in serum of patients with coronary artery diesease. Knockout reduced atherosclerosis in mice	[20,21]
FASLG	2.43	0.92		
IFNA2	1.53	0.67		
IFNG	1.40	0.05	Displays both pro- and anti-atherogenic functions	[27]
IL10RA	3.94	2.16		
IL10RB	1.21	1.27		
IL13	0.44	0.48	IL-13 promotes plaque stability and reduces VCAM-1-mediated monocyte recruitment	[28]
IL15	2.81	0.91	Vaccination against IL15 reduced atherosclerosis in mice	[29]
IL16	7.46	5.86		
IL17A	0.38	0.13	Antibody therapy reduced atherosclerosis in mice	[30]
IL17C	0.33	0.38		
IL17F	0.76	0.52		
IL1A	17.39	11.08	Antibody therapy reduced atherosclerosis in mice	[31]
IL1B	7.26	7.01	Knockout reduced atherosclerosis in mice	[32]
IL1R1	1.59	0.76	Promotes outward remodeling and plaque stabilization in advanced lesions	[33]
IL1RN	N/A	N/A		
IL21	0.59	0.46		
IL27	0.24	0.29	Knockout increased atherosclerosis in mice	[34]
IL3	1.16	0.43		
IL33	0.23	0.02	IL-33 treatment reduces atherosclerosis in mice	[35]
IL5	0.63	0.11	IL5 bonemarrow knockout increased atherosclerosis in mice	[36]
IL5RA	N/A	N/A		
IL7	44.32	5.17	Upregulates cell adhesion molecules and MCP-1 expression in endothelial cells, and reducing cholesterol lowers IL-7 expression	[37]
CXCL8	5.21	2.75	Found in human atheromas	[38]
IL9	1.27	0.58		
IL9R	N/A	N/A		
LTA	1.14	1.75		
LTB	42.52	29.86	LTB receptor knockout reduces plaque size	[39]
MIF	0.57	0.59	Knockout reduces atherosclerosis in mice	[40]
NAMPT	1.27	0.55	Knockdown redcues atherosclerosis and promotes cholsterol eflux in mice	[41]
OSM	3.66	1.47	Expressed in atherosclerosis, and knockout of it's receptor reduces atherosclerosis in mice	[42,43]
SPP1	2.19	N/A	Knockout reduced atherosclerosis in female mice, elevated in patients with CAD and associated with rapid plaque progression in instent restensosis	[44]
TNF	N/A	N/A	Knockout reduces atherosclerosis in mice	[45]
TNFRSF11B	1.64	0.27	Several polymorphisms were elevated in carotid plaques, and were associated with eleveted serum level siand unstable plaques	[46]
TNFSF10	36.25	3.25	Induce inflammatory gene expression in human endothelial cells. Knockout increases early atherosclerosis in mice	[47,48]
TNFSF11	0.45	0.23	Elevated in *ApoE-/-* mice, potential marker for detecting atherosclerosis	[49]
TNFSF13	0.97	1.61	Expressed in atherosclerosis, and elevated in patients with CAD	[50]
TNFSF13B	131.60	1.92	Upregulated in human atherosclerosis	[51]
TNFSF4	0.28	0.54		
VEGFA	0.41	0.69	Overexpression increased plaque size in mice	[52]

Supplemental Table 2. Shown are fold changes above untreatead controls normalized to β-actin. mRNA levels were determined using the Human Inflammatory Cytokines and Receptors RT2 Profiler PCR Array (Cat. No. 330231 PAHS-011ZA; QIAGEN). Note: N/A: Not Available, no mRNA was detected

Gene Name	IL-1β	IL-1β + PF-271	Potential Role in Atherosclerosis	References
AIMP1	1.28	0.77		
BMP2	3.97	0.71		
C5	0.73	4.29		
CCL1	1.28	0.72		
CCL11	2.25	2.00		
CCL13	2.35	0.54		
CCL15	0.14	0.91		
CCL16	0.59	1.16		
CCL17	N/A	39.67	Upregulatead in human atherosclerotic plaques	[1]
CCL2	28.64	6.54	Found in both mouse and human atherosclerosis. Knockout reduced atherosclerosis in *Ldlr-/-* mice	[2-4]
CCL20	121.10	2.22	Elevated levels found in hypercholesterolemic patients,	[5]
CCL22	N/A	N/A		
CCL23	0.24	2.10		
CCL24	2.33	0.79		
CCL26	1.57	1.12		
CCL3	28.05	1.22	Expressed in human atherosclerosis	[6]
CCL4	0.46	N/A	Expressed in human atherosclerosis	[6]
CCL5	51.63	2.81	Expressed in both mouse and human atherosclerosis	[7,8]
CCL7	25.81	0.82		
CCL8	1.83	5.50		
CCR1	1.21	0.75	Knockout increased atherosclerosis in mice	[9]
CCR2	1.51	0.79	Knockout reduced atherosclerosis in mice	[10,11]
CCR3	0.45	0.78		
CCR4	4.82	2.35		
CCR5	0.36	0.95	Knockout reduced atherosclerosis in mice	[9,12]
CCR6	0.81	3.81	Knockout reduced atherosclerosis in mice	[13]
CCR8	1.53	1.14		
CD40LG	2.10	1.29		
CSF1	3.61	5.03	Knockout reduced atherosclerosis in mice. Reduced inflammation and increased macropahge apoptosis in lesions	[14,15]
CSF2	98.36	17.03	Knockout reduced atherosclerosis in mice	[16]
CSF3	107.63	1.97		
CX3CL1	9.06	1.55	Knockout reduced atherosclerosis in mice	[17]
CX3CR1	0.18	0.28	Knockout reduced atherosclerosis in mice	[18,19]
CXCL1	19.70	8.11	Increased in serum of patients with coronary artery diesease. Knockout reduced atherosclerosis in mice	[20,21]
CXCL10	3.32	1.91	Expressed in human atherosclerosis. Knockout reduced atherosclerosis in mice	[22,23
CXCL11	6.41	0.92		
CXCL12	1.14	0.29	Found in human atherosclerosis, and reduced serum levels in coronary artery disease patients and old *ApoE-/-* mice	[24-26]
CXCL13	0.80	0.80		
CXCL2	17.75	9.65		
CXCL3	28.44	32.22		
CXCL5	2.06	7.11		
CXCL6	37.27	0.92		
CXCL9	1.16	0.90		
CXCR1	0.84	0.21		
CXCR2	1.15	0.46	Increased in serum of patients with coronary artery diesease. Knockout reduced atherosclerosis in mice	[20,21]
FASLG	1.91	3.51		
IFNA2	0.06	2.48		
IFNG	1.40	1.25	Displays both pro- and anti-atherogenic functions	[27]
IL10RA	5.28	5.03		
IL10RB	1.01	0.38		
IL13	1.92	0.92	IL-13 promotes plaque stability and reduces VCAM-1-mediated monocyte recruitment	[28]
IL15	1.75	0.28	Vaccination against IL15 reduced atherosclerosis in mice	[29]
IL16	2.64	0.17		
IL17A	0.76	0.10	Antibody therapy reduced atherosclerosis in mice	[30]
IL17C	1.27	0.08		
IL17F	1.87	0.84		
IL1A	21.86	1.16	Antibody therapy reduced atherosclerosis in mice	[31]
IL1B	268.73	1.46	Knockout reduced atherosclerosis in mice	[32]
IL1R1	1.52	0.02	Promotes outward remodeling and plaque stabilization in advanced lesions	[33]
IL1RN	N/A	N/A		
IL21	0.35	0.24		
IL27	1.01	0.66	Knockout increased atherosclerosis in mice	[34]
IL3	2.68	1.41		
IL33	0.28	0.14	IL-33 treatment reduces atherosclerosis in mice	[35]
IL5	2.79	0.39	IL5 bonemarrow knockout increased atherosclerosis in mice	[36]
IL5RA	N/A	N/A		
IL7	41.64	5.03	Upregulates cell adhesion molecules and MCP-1 expression in endothelial cells, and reducing cholesterol lowers IL-7 expression	[37]
CXCL8	20.39	1.43	Found in human atheromas	[38]
IL9	1.53	0.18		
IL9R	N/A	N/A		
LTA	1.17	0.62		
LTB	2.53	1.16	LTB receptor knockout reduces plaque size	[39]
MIF	1.05	1.07	Knockout reduces atherosclerosis in mice	[40]
NAMPT	5.98	0.13	Knockdown redcues atherosclerosis and promotes cholsterol eflux in mice	[41]
OSM	2.31	9.06	Expressed in atherosclerosis, and knockout of it's receptor reduces atherosclerosis in mice	[42,43]
SPP1	0.21	2.69	Knockout reduced atherosclerosis in female mice, elevated in patients with CAD and associated with rapid plaque progression in instent restensosis	[44]
TNF	N/A	N/A	Knockout reduces atherosclerosis in mice	[45]
TNFRSF11B	9.92	0.27	Several polymorphisms were elevated in carotid plaques, and were associated with eleveted serum level siand unstable plaques	[46]
TNFSF10	2.73	0.77	Induce inflammatory gene expression in human endothelial cells. Knockout increases early atherosclerosis in mice	[47,48]
TNFSF11	0.62	0.78	Elevated in *ApoE-/-* mice, potential marker for detecting atherosclerosis	[49]
TNFSF13	0.97	0.66	Expressed in atherosclerosis, and elevated in patients with CAD	[50]
TNFSF13B	3.51	2.06	Upregulated in human atherosclerosis	[51]
TNFSF4	0.34	0.14		
VEGFA	1.31	1.82	Overexpression increased plaque size in mice	[52]

Supplemental Table 3: List of antibodies used.

Antibody	Catalog #	Vendor	Use	Concentration
FAK	05-537	Millipore	Western Blot<br>Cell Staining	1:2000<br>1:200
p-ERK1/2	04-797	Millipore	Western Blot	1:5000
p-p38	09-272	Millipore	Western Blot	1:2000
αSMA	ABT1487	Millipore	Tissue Staining	1:200
p38	MABS1754	Millipore	Western Blot	1:2000
VCAM-1	SC-8304	Santa Cruz	Western Blot<br>Tissue Staining	1:2000<br>1:200
ICAM-1	SC-18853	Santa Cruz	Western Blot	1:2000
E-Selectin	SC-14011	Santa Cruz	Western Blot	1:1000
IκBα	SC-371	Santa Cruz	Western Blot	1:2000
vWF	SC-271409	Santa Cruz	Tissue Staining	1:200
Src	sc-18	Santa Cruz	Western Blot	1:2000
JNK	sc-7345	Santa Cruz	Western Blot	1:2000
ERK	sc-154	Santa Cruz	Western Blot	1:2000
β-actin	A5316	Sigma-Aldrich	Western Blot	1:5000
αSMA	202M-9	Sigma-Aldrich	Tissue Staining	1:200
pY418 Src	21013	Cell Signal	Western Blot	1:2000
Pyk2	3480	Cell Signal	Western Blot	1:2000
pS536 p65	3033	Cell Signal	Western Blot	1:2000
p-JNK	9251	Cell Signal	Western Blot	1:2000
VCAM-1	ab134047	Abcam	Cell Staining	1:200
CD68	ab31630	Abcam	Tissue Staining	1:200
pY402 Pyk2	MAB6210	R&D Systems	Western Blot	1:2000
pY397 FAK	44-624G	Life Technologies	Western Blot	1:2000
pY576 FAK	700013	Life Technologies	Tissue Staining	1:200
Goat anti-Rabbit HRP	31460	Life Technologies	Western Secondary	1:5000
Goat anti-Mouse HRP	31430	Life Technologies	Western Secondary	1:5000
Goat anti-Mouse Alexa Fluor 488	A-11029	Life Technologies	Cell Staining<br>Tissue Staining	1:1000
Goat anti-Mouse Alexa Fluor 594	A-11032	Life Technologies	Cell Staining<br>Tissue Staining	1:1000<br>1:1000
Goat anti-Rabbit Alexa Fluor 488	A-11034	Life Technologies	Cell Staining<br>Tissue Staining	1:1000<br>1:1000
Goat anti-Rabbit Alexa Fluor 594	R37117	Life Technologies	Cell Staining<br>Tissue Staining	1:1000<br>1:1000

Supplemental Table 4: RT-qPCR Primers

**Primer**	**Sequence (5’ -> 3’)**
VCAM-1 Forward	AGTTGAAGGATGCGGGAGTA
VCAM-1 Reverse	AGAGCACGAGAAGCTCAGGA
ICAM-1 Forward	GTGCTATTCAAACTGCCC
ICAM-1 Reverse	GTAGGGTAAGGTTCTTGC
E-Selectin Forward	GCAGCAAGAAGAAGCTTG
E-Selectin Reverse	GGATTCCAGGGCTGTACA
CCL2 Forward	ATGCAATCAATGCCCCAG
CCL2 Reverse	GAATCCTGAACCCACTTCT
CXCL11 Forward	TGTGAAGGGCATGGCTAT
CXCL11 Reverse	GGCTTTCTCAATATCTGC
IP-10 Forward	GCCATTCTGATTTGCTGC
IP-10 Reverse	TGATCTCAACACGTGGAC
GAPDH Forward	GGCGCTGAGTACGTCGTGGAGTCCA
GAPDH Reverse	AAAGTTGTCATGGATGACCTTGG

Supplemental Table References

1. Greaves, D. R. *et al.* Linked chromosome 16q13 chemokines, macrophage-derived chemokine, fractalkine, and thymus- and activation-regulated chemokine, are expressed in human atherosclerotic lesions. *Arterioscler Thromb Vasc Biol* **21**, 923-929 (2001).

2. Rayner, K., Van Eersel, S., Groot, P. H. & Reape, T. J. Localisation of mrna for je/mcp-1 and its receptor ccr2 in atherosclerotic lesions of the apoe knockout mouse. *J Vasc Res* **37**, 93-102 (2000).

3. Nelken, N. A., Coughlin, S. R., Gordon, D. & Wilcox, J. N. Monocyte chemoattractant protein-1 in human atheromatous plaques. *J Clin Invest* **88**, 1121-1127 (1991).

4. Gu, L. *et al.* Absence of monocyte chemoattractant protein-1 reduces atherosclerosis in low density lipoprotein receptor-deficient mice. *Mol Cell* **2**, 275-281 (1998).

5. Calvayrac, O. *et al.* Ccl20 is increased in hypercholesterolemic subjects and is upregulated by ldl in vascular smooth muscle cells: Role of nf-kappab. *Arterioscler Thromb Vasc Biol* **31**, 2733-2741 (2011).

6. Wilcox, J. N., Nelken, N. A., Coughlin, S. R., Gordon, D. & Schall, T. J. Local expression of inflammatory cytokines in human atherosclerotic plaques. *J Atheroscler Thromb* **1 Suppl 1**, S10-13 (1994).

7. Krohn, R. *et al.* Y-box binding protein-1 controls cc chemokine ligand-5 (ccl5) expression in smooth muscle cells and contributes to neointima formation in atherosclerosis-prone mice. *Circulation* **116**, 1812-1820 (2007).

8. Pattison, J. M., Nelson, P. J., Huie, P., Sibley, R. K. & Krensky, A. M. Rantes chemokine expression in transplant-associated accelerated atherosclerosis. *J Heart Lung Transplant* **15**, 1194-1199 (1996).

9. Braunersreuther, V. *et al.* Ccr5 but not ccr1 deficiency reduces development of diet-induced atherosclerosis in mice. *Arterioscler Thromb Vasc Biol* **27**, 373-379 (2007).

10. Boring, L., Gosling, J., Cleary, M. & Charo, I. F. Decreased lesion formation in ccr2-/- mice reveals a role for chemokines in the initiation of atherosclerosis. *Nature* **394**, 894-897 (1998).

11. Dawson, T. C., Kuziel, W. A., Osahar, T. A. & Maeda, N. Absence of cc chemokine receptor-2 reduces atherosclerosis in apolipoprotein e-deficient mice. *Atherosclerosis* **143**, 205-211 (1999).

12. Quinones, M. P. *et al.* Cc chemokine receptor 5 influences late-stage atherosclerosis. *Atherosclerosis* **195**, e92-103 (2007).

13. Wan, W. *et al.* Genetic deletion of chemokine receptor ccr6 decreases atherogenesis in apoe-deficient mice. *Circ Res* **109**, 374-381 (2011).

14. Qiao, J. H. *et al.* Role of macrophage colony-stimulating factor in atherosclerosis: Studies of osteopetrotic mice. *Am J Pathol* **150**, 1687-1699 (1997).

15. Shaposhnik, Z., Wang, X. & Lusis, A. J. Arterial colony stimulating factor-1 influences atherosclerotic lesions by regulating monocyte migration and apoptosis. *J Lipid Res* **51**, 1962-1970 (2010).

16. Subramanian, M., Thorp, E. & Tabas, I. Identification of a non-growth factor role for gm-csf in advanced atherosclerosis: Promotion of macrophage apoptosis and plaque necrosis through il-23 signaling. *Circ Res* **116**, e13-24 (2015).

17. Teupser, D. *et al.* Major reduction of atherosclerosis in fractalkine (cx3cl1)-deficient mice is at the brachiocephalic artery, not the aortic root. *Proc Natl Acad Sci U S A* **101**, 17795-17800 (2004).

18. Combadiere, C. *et al.* Decreased atherosclerotic lesion formation in cx3cr1/apolipoprotein e double knockout mice. *Circulation* **107**, 1009-1016 (2003).

19. Lesnik, P., Haskell, C. A. & Charo, I. F. Decreased atherosclerosis in cx3cr1-/- mice reveals a role for fractalkine in atherogenesis. *J Clin Invest* **111**, 333-340 (2003).

20. Breland, U. M. *et al.* A potential role of the cxc chemokine groalpha in atherosclerosis and plaque destabilization: Downregulatory effects of statins. *Arterioscler Thromb Vasc Biol* **28**, 1005-1011 (2008).

21. Boisvert, W. A. *et al.* Up-regulated expression of the cxcr2 ligand kc/gro-alpha in atherosclerotic lesions plays a central role in macrophage accumulation and lesion progression. *Am J Pathol* **168**, 1385-1395 (2006).

22. Mach, F. *et al.* Differential expression of three t lymphocyte-activating cxc chemokines by human atheroma-associated cells. *J Clin Invest* **104**, 1041-1050 (1999).

23. Heller, E. A. *et al.* Chemokine cxcl10 promotes atherogenesis by modulating the local balance of effector and regulatory t cells. *Circulation* **113**, 2301-2312 (2006).

24. Abi-Younes, S. *et al.* The stromal cell-derived factor-1 chemokine is a potent platelet agonist highly expressed in atherosclerotic plaques. *Circ Res* **86**, 131-138 (2000).

25. Damas, J. K. *et al.* Stromal cell-derived factor-1alpha in unstable angina: Potential antiinflammatory and matrix-stabilizing effects. *Circulation* **106**, 36-42 (2002).

26. Xu, Q. *et al.* Impaired cxcr4 expression and cell engraftment of bone marrow-derived cells from aged atherogenic mice. *Atherosclerosis* **219**, 92-99 (2011).

27. Harvey, E. J. & Ramji, D. P. Interferon-gamma and atherosclerosis: Pro- or anti-atherogenic? *Cardiovasc Res* **67**, 11-20 (2005).

28. Cardilo-Reis, L. *et al.* Interleukin-13 protects from atherosclerosis and modulates plaque composition by skewing the macrophage phenotype. *EMBO Mol Med* **4**, 1072-1086 (2012).

29. van Es, T. *et al.* Il-15 aggravates atherosclerotic lesion development in ldl receptor deficient mice. *Vaccine* **29**, 976-983 (2011).

30. Erbel, C. *et al.* Il-17a influences essential functions of the monocyte/macrophage lineage and is involved in advanced murine and human atherosclerosis. *J Immunol* **193**, 4344-4355 (2014).

31. Tissot, A. C. *et al.* A vlp-based vaccine against interleukin-1alpha protects mice from atherosclerosis. *Eur J Immunol* **43**, 716-722 (2013).

32. Kirii, H. *et al.* Lack of interleukin-1beta decreases the severity of atherosclerosis in apoe-deficient mice. *Arterioscler Thromb Vasc Biol* **23**, 656-660 (2003).

33. Alexander, M. R. *et al.* Genetic inactivation of il-1 signaling enhances atherosclerotic plaque instability and reduces outward vessel remodeling in advanced atherosclerosis in mice. *J Clin Invest* **122**, 70-79 (2012).

34. Hirase, T. *et al.* Interleukin 27 inhibits atherosclerosis via immunoregulation of macrophages in mice. *Am J Physiol Heart Circ Physiol* **305**, H420-429 (2013).

35. Miller, A. M. *et al.* Il-33 reduces the development of atherosclerosis. *J Exp Med* **205**, 339-346 (2008).

36. Binder, C. J. *et al.* Il-5 links adaptive and natural immunity specific for epitopes of oxidized ldl and protects from atherosclerosis. *J Clin Invest* **114**, 427-437 (2004).

37. Li, R. *et al.* Interleukin-7 induces recruitment of monocytes/macrophages to endothelium. *Eur Heart J* **33**, 3114-3123 (2012).

38. Wang, N. *et al.* Interleukin 8 is induced by cholesterol loading of macrophages and expressed by macrophage foam cells in human atheroma. *The Journal of biological chemistry* **271**, 8837-8842 (1996).

39. Grandoch, M. *et al.* Deficiency in lymphotoxin beta receptor protects from atherosclerosis in apoe-deficient mice. *Circ Res* **116**, e57-68 (2015).

40. Pan, J. H. *et al.* Macrophage migration inhibitory factor deficiency impairs atherosclerosis in low-density lipoprotein receptor-deficient mice. *Circulation* **109**, 3149-3153 (2004).

41. Li, S. *et al.* Nampt knockdown attenuates atherosclerosis and promotes reverse cholesterol transport in apoe ko mice with high-fat-induced insulin resistance. *Sci Rep* **6**, 26746 (2016).

42. Albasanz-Puig, A. *et al.* Oncostatin m is expressed in atherosclerotic lesions: A role for oncostatin m in the pathogenesis of atherosclerosis. *Atherosclerosis* **216**, 292-298 (2011).

43. Zhang, X. *et al.* Oncostatin m receptor beta deficiency attenuates atherogenesis by inhibiting jak2/stat3 signaling in macrophages. *J Lipid Res* **58**, 895-906 (2017).

44. Mazzone, A. *et al.* Osteopontin plasma levels and accelerated atherosclerosis in patients with cad undergoing pci: A prospective clinical study. *Coron Artery Dis* **22**, 179-187 (2011).

45. Ohta, H. *et al.* Disruption of tumor necrosis factor-alpha gene diminishes the development of atherosclerosis in apoe-deficient mice. *Atherosclerosis* **180**, 11-17 (2005).

46. Straface, G. *et al.* Assessment of the genetic effects of polymorphisms in the osteoprotegerin gene, tnfrsf11b, on serum osteoprotegerin levels and carotid plaque vulnerability. *Stroke* **42**, 3022-3028 (2011).

47. Li, J. H., Kirkiles-Smith, N. C., McNiff, J. M. & Pober, J. S. Trail induces apoptosis and inflammatory gene expression in human endothelial cells. *J Immunol* **171**, 1526-1533 (2003).

48. Watt, V., Chamberlain, J., Steiner, T., Francis, S. & Crossman, D. Trail attenuates the development of atherosclerosis in apolipoprotein e deficient mice. *Atherosclerosis* **215**, 348-354 (2011).

49. Tabibiazar, R., Wagner, R. A., Deng, A., Tsao, P. S. & Quertermous, T. Proteomic profiles of serum inflammatory markers accurately predict atherosclerosis in mice. *Physiol Genomics* **25**, 194-202 (2006).

50. Sandberg, W. J. *et al.* The tumour necrosis factor superfamily ligand april (tnfsf13) is released upon platelet activation and expressed in atherosclerosis. *Thromb Haemost* **102**, 704-710 (2009).

51. Turpeinen, H. *et al.* Proprotein convertases in human atherosclerotic plaques: The overexpression of furin and its substrate cytokines baff and april. *Atherosclerosis* **219**, 799-806 (2011).

52. Heinonen, S. E. *et al.* The effects of vegf-a on atherosclerosis, lipoprotein profile, and lipoprotein lipase in hyperlipidaemic mouse models. *Cardiovasc Res* **99**, 716-723 (2013).
